# Synthesis, antimicrobial and molecular docking study of structural analogues of 3-((5-(dimethylcarbamoyl)pyrrolidin-3-yl)thio)-6-(1-hydroxyethyl)-4-methyl-7-oxo-1-azabicyclo[3.2.0]heptane-2-carboxylic acid

**DOI:** 10.1371/journal.pone.0278684

**Published:** 2022-12-27

**Authors:** Saharish Khaliq, Mohsin Abbas Khan, Irshad Ahmad, Imtiaz Ahmad, Javed Ahmed, Farhat Ullah

**Affiliations:** 1 Department of Pharmaceutical Chemistry, Faculty of Pharmacy, The Islamia University of Bahawalpur, Bahawalpur, Pakistan; 2 Department of Medicinal Chemistry, College of Pharmacy, University of Minnesota, Minneapolis, Minnesota, United States of America; Aligarh Muslim University, INDIA

## Abstract

The goal of the current work was to create structural analogues of a beta lactam antibiotic that might be possibly effective against bacterial resistant strains. FTIR, ^1^H NMR, ^13^C NMR, and CHNS analyses were used to perform the spectroscopic study on the compounds M_1–8_. The effects of the aforementioned substances on gram-positive and gram-negative bacterial strains were investigated. Most of the eight compounds had antibacterial activity that was lower than or equivalent to that of the original medication, but two molecules, M_2_ and M_3_, surprisingly, had stronger antibacterial activity. The findings of synthesized analogues against alpha-glucosidase and DPPH inhibition were found to be modest, whereas M_2_, M_3_, and M_7_ strongly inhibited the urease. To comprehend the potential mode of action, a molecular docking research was conducted against urease and -amylase. The research may help in the quest for novel chemical compounds that would be effective against bacteria that are resistant to antibiotics.

## 1. Introduction

Worldwide, there is an alarming increase in bacterial resistance that threatens the effectiveness of currently available antibiotics. Therefore, it is still crucial to create new, powerful antibacterial agents. Antibiotic-resistant bacteria are an international issue that raises healthcare expenditures and death. The biggest issue that humanity is now facing is bacterial resistance. Therefore, the time has come to develop new compounds with enhanced antibacterial activity and a novel mode of action [1]. Antimicrobial resistance has harmed human health and had an impact on the economy as a whole [2]. Methicillin-resistant *Staphylococcus aureus* (MRSA), carbapenem-resistant *Enterobacteriaceae* (CRE), multidrug-resistant *Mycobacterium tuberculosis* (MDR-TB), vancomycin-resistant *Enterococcus* (VRE), and multidrug-resistant *Streptococcus pneumonia* infections are particularly difficult to treat. To combat bacterial strains that are resistant to antibiotics, this has compelled attention in the creation of novel and powerful antibacterial agents [1]. The largest danger to world health is the rise in multidrug-resistant microbial strains, which is expected to result in >10 million fatalities by 2055. Antibacterial medications from the carbapenem family are a crucial subset of last-resort care for illnesses brought on by bacteria with antibiotic resistance. A significant global danger that is predicted to result in over 10 million fatalities by 2055 is multidrug resistance [3]. Pathogenic bacteria continually develop their resistance mechanisms, which poses a severe challenge to the management of infectious diseases [4]. Due to its low absorption, carbapenem is a subclass of medications that is only used as a last option to treat drug-resistant bacteria. It is commercially accessible as injectables. The compound 3-((5-(dimethylcarbamoyl)pyrrolidin-3-yl)thio) -6- (1-hydroxyethyl) (1-hydroxyethyl) -4-methyl -7-oxo -1-azabicyclo [3.2.0] Since heptane-2 carboxylic acid is the preferred medication and is accessible as an injectable, it is crucial to explore multiple strategies for meropenem oral drug administration. A prodrug strategy might be used to compensate for carbapenem’s limited availability (meropenem). One strategy for creating a prodrug is to hide undesirable drug characteristics such drug instability, low bioavailability, and lack of site-specificity [5]. A common strategy for improving oral absorption in this class of drugs is the production of ester as the prodrug. The oral absorption of such medications can be improved by adding lipophilic moieties at the carbapenem C-3 and pyrrolidine N-1 sites [3]. Strong antibacterial capabilities are handled by all carbapenems since they all include pyrrolidine-3-yl thio groups at the C-2 position in their basic structure. It referred to them as "final line agents" or "antibiotics of last resort" due to their increased effectiveness [6]. Thienamycin, the first carbapenem to be found, was derived from *Streptomyces cattleya* [7]. One of the most often used medications for illnesses that pose a serious risk of death is meropenem, which was developed in the late 1980s. The quantity of beta-lactamases and dehydropeptidase does not cause meropenem to become inactive [8]. It is effective against extended-spectrum beta-lactamase, gram-positive and gram-negative bacteria (ESBL). Its mode of action centers on the suppression of cell wall synthesis, which leads to ultimate cellular death. The compound The preferred drug of choice for bacterial meningitis, severe skin infections, febrile neutropenia, respiratory infections, and urinary tract infections is 3-((5-(dimethylcarbamoyl)pyrrolidin-3-yl)thio) -6-(1-hydroxyethyl) -4-methyl -7-oxo1-azabicycloheptane-2-carboxylic acid. It is recommended for the treatment of community-acquired pneumonia, pulmonary exacerbations, and gynecological infections [9]. New derivatives of these substances have been found as a result of the rise in drug resistance. Ester derivatives showed improved biological and pharmacological capabilities, according to the literature study. The parent medicine, nalidixic acid, and its ester derivatives have broad antibacterial action against bacteria like Aeromonas hydrophila and Streptococcus pyrogens that are resistant to nalidixic acid in its purest form. Some new beta-lactam compounds shown outstanding antioxidant activity [10]. In addition to being tested for antibacterial activity, newly synthesized 4-alkylidene-b-lactam derivatives shown outstanding radical scavenging activity [11]. Seven out of 17 new quinolones demonstrated a substantial amylase inhibition in studies, indicating that the proline ring is what inhibits amylase [12]. The proline ring is a heterocyclic molecule that has a variety of properties, including antimicrobial, antioxidant, anti-carcinogen, anti-HIV, anti-inflammatory, and -amylase inhibitory active moiety [12]. Urinary tract infections and Helicobacter pylori are the two most dangerous illnesses associated with urease activity [13]. Meropenem is the recommended medication for UTIs, thus derivatives generated in the presence of urease inhibition can demonstrate if innovative compounds have greater capacity to withstand urease inhibition than the original substance [9].

The glycerol esters and diesters of sucrose have stronger antibacterial properties [14], and a number of esters derivatives also have antifungal, anticancer, anti-inflammatory, analgesic, and anesthetic properties When indomethacin is reacted with alcohol and phenol in the presence of 4-dimethylaminopyridine and dicyclohexylcarbodiimide (DCC), ester derivatives of indomethacin are created, which inhibit cox-2 enzymes more effectively and have no gastrointestinal adverse effects [15]. Our research focused on producing 3- ((5- (dimethylcarbamoyl) pyrrolidin-3-yl) thio)-6- ester derivatives of (1-hydroxyethyl) -4-methyl-7-oxo-1-azabicyclo [3.2.0] heptane-2-carboxylic acid has better pharmacokinetic characteristics and biological activity.

## 2. Experimental

### 2.1 Synthesis

**Fig 1:**
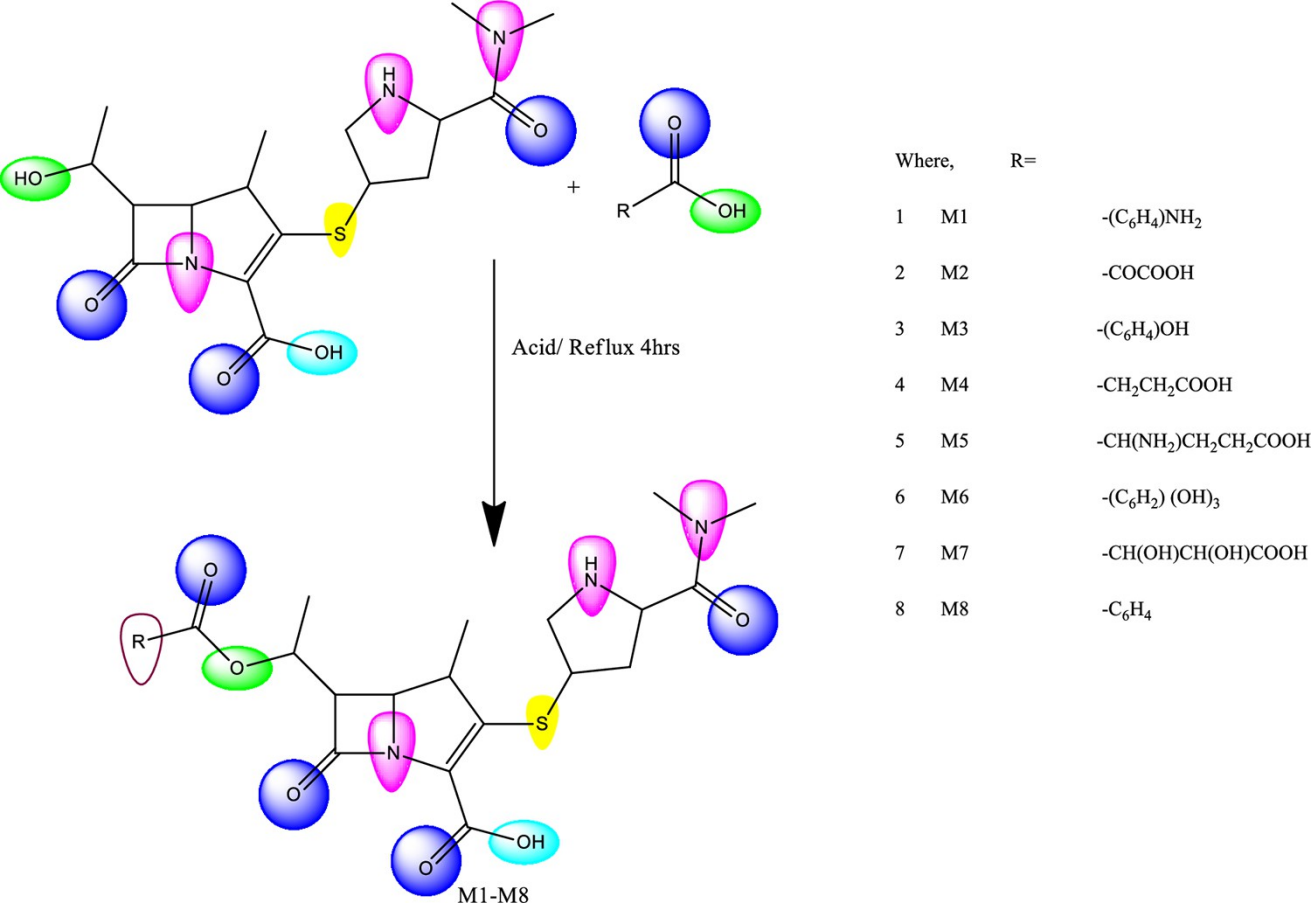
Schematic diagram for synthesis of ester derivatives of 3-((5-(dimethylcarbamoyl) pyrrolidin-3-yl) thio)-6-(1-hydroxyethyl)-4-methyl-7-oxo-1-azabicyclo[3.2.0] heptane-2-carboxylic acid.

**Fig 2:**
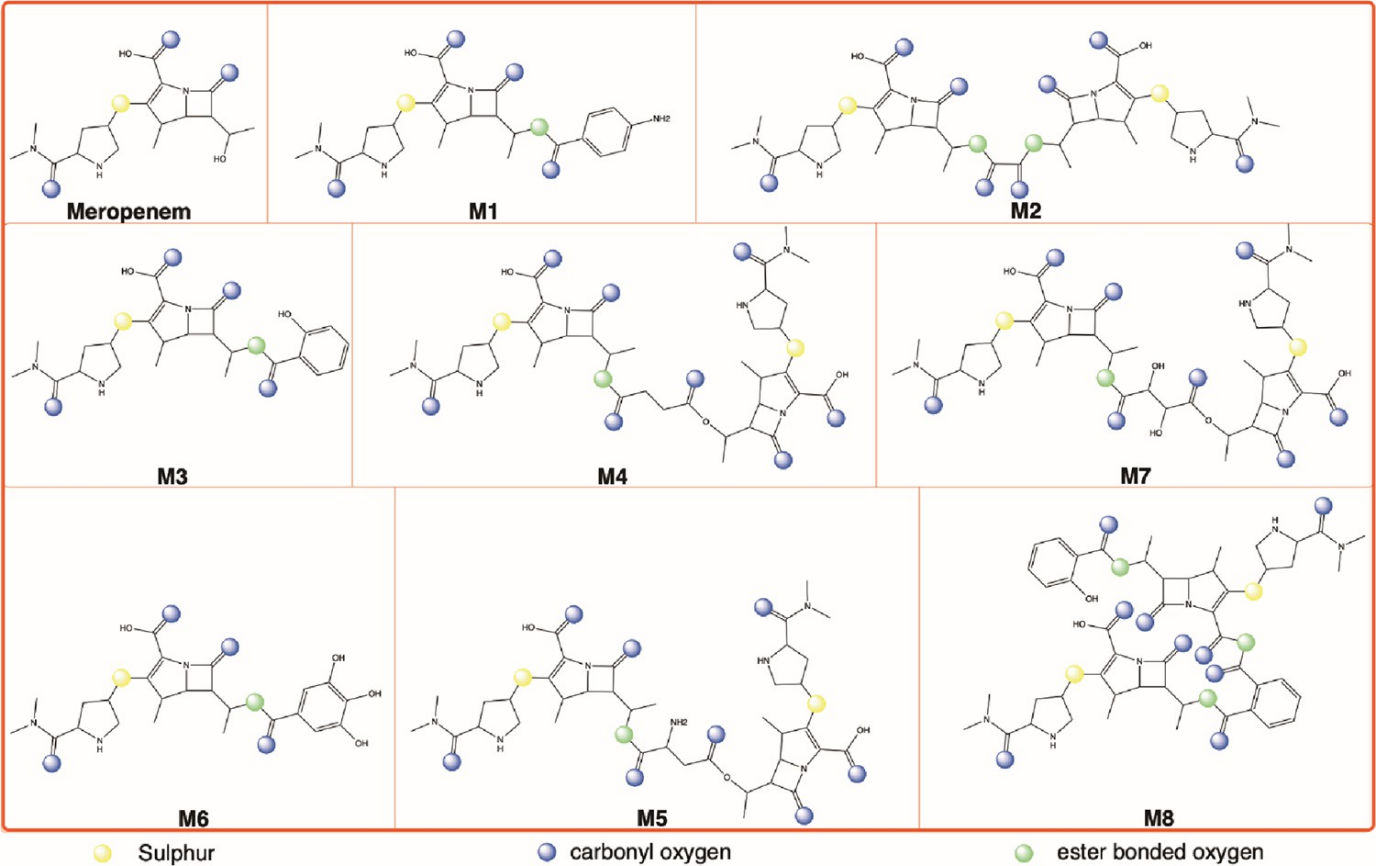
Chemical structures of Meropenem and its synthesized derivatives M_1_-M_8_.

Equimolar mixtures of the appropriate carboxylic acid and meropenem were added to a round-bottomed flask, followed by the addition of 30 ml of 99.8% pure ethanol (34852-M Sigma Aldrich®) and concentrated HCl, and refluxed for 4 hours on the water bath to create M_**1**_-_**8**_ (Figs 1 and 2). After the reaction was finished, the mixture was held at room temperature before being filtered and evaporated using a rotary process at a lower temperature and pressure. The crude product was recrystallized with the help of the ethanol.

### 2.2 Spectroscopic and elemental analysis of M_1_-_8_ compounds

It was examined if synthetic compounds were soluble in ethanol, methanol, water, chloroform, and DMSO. The melting point apparatus developed by Gallen Kamp was used to determine the melting point. The CHNS analyzer is used to determine the amount of carbon, hydrogen, and nitrogen. Bruker FTIR (Tensor model 27) and NMR 500 MHz are utilized for spectroscopic analysis.

### 2.3 Biological evaluation

Biological evaluation of M_1_ to M_8_ included antibacterial evaluation, enzyme inhibition assay, and antioxidant study.

#### 2.3.1 Antibacterial activity

Gram-positive and gram-negative bacteria were tested using the agar well diffusion technique, as reported in the literature [16] (e.g. *Escherichia coli*, *Stenotrophomonas maltophilia*, *Bacillus megatarium*, *Micrococcus luteus*, *Bacillus subtilis*, *Staphylococcus aureus*, *and Serratia marcescens* were obtained from the Department of Microbiology at the Islamia University of Bahawalpur in Pakistan. As a positive control, meropenem was utilized, and as a negative control, 5% DMSO.

#### 2.3.2 Enzyme inhibition assays

The literature-recommended procedures for urease enzyme inhibition and alpha-amylase enzyme inhibition experiments were followed with a few minor modifications, and the findings were represented as mg. Eq/gram of the corresponding positive control [12, 17].

#### 2.3.3 Antioxidant activity

Using the procedure outlined in the literature, DPPH (1,1-diphenyl-2-picryl hydrazyl) was used to test the scavenging capability of derivatives [18]. The findings were computed using the following formula and expressed as a percentage inhibition of free radicals:

$$\%\;inhibition=\frac{control\;solution-sample\;solution}{control\;solution}x\;100$$

### 2.4 Molecular docking

#### 2.4.1 Ligand preparation

Using the semi-empirical quantum mechanical technique PM3, the structures of all meropenem derivatives were constructed and their geometries were optimized. ChemDraw 12.0’s SDF format was used to generate the 3D conformers of the tested compounds for docking. The PyRx 0.08 application imported all tested substances into OpenBabel [19], where they were subjected to energy reduction, in order to change the docking file format from sdf to pdbqt format. With the use of a universal force field (UFF) and an energy difference of less than 0.01 kcal/mol, the conjugate gradient technique was employed to reduce energy. The minimized compounds were then transferred to PDBQT format for further examination.

#### 2.4.2 Protein structure preparation

The crystal structures of the enzymes urease (PDB ID: 4H9M) [22] and human pancreatic -amylase (PDB code: 5E0F) [20] were retrieved from Protein Data Bank. The protein’s built-in ligands were taken out of the crystal structure. Water molecules were eliminated from the protein by adding polar hydrogens and Kollman charges. In order to make analysis easier at a later stage of the simulation, final files were saved in PDBQT format.

#### 2.4.3 Protocol of docking study

Auto-Dock version 4.2 was used to perform the docking study. According to the macromolecular target site, the Auto Grid component, for instance, pre-calculates a three-dimensional grid of interaction energies using the AMBER force field. Automated docking tests were carried out to measure the binding free energy of the inhibitor. The most effective conformers were selected using the genetic algorithm. A variety of parameters, including population size and run counts, were specified. The crossover rate was set at 0.8, while the mutation rate was set at 0.02. The positional RMSD (Root Mean Square Deviation) of these findings varied by less than 0.5, and the resulting complex structures had the lowest binding energies.

### 2.5 ADME prediction

The choice of an agent as a drug is heavily influenced by the physical and molecular properties of substances. The Swiss ADME web server (http://www.swissadme.ch) was used to examine the molecular characteristics of new derivatives in order to confirm their potential as therapeutic target ligands [21]. In order to examine pharmacokinetics factors like as absorption, lipophilicity, and water solubility, each drug was added to the online server as a smile format. The topological polar surface area was used to compute the percentage of absorption (%ABS) of new derivatives:%ABS = 109-(0.345TPSA).

## 3. Results

### 3.1 Spectroscopic analysis

#### 3.1.1 M1: 6- (1-((4-aminobenzoyl)oxy)ethyl)-3-((5 (dimethylcarbamoyl) pyrrolidin-3-yl)thio)- 4 methyl-7oxo 1-azabicyclo[3.2.0]hept-2-ene-2-carboxylic acid

Yield (71%); m.p 140–142°C; insoluble in chloroform but soluble in ethanol, methanol, DMSO and water Molecular Formula: C_24_H_30_N_4_O_6_S and Molecular weight: 502.59 gm/Mol Elemental analysis (calculated) for C_24_H_30_N_4_O_6_S: C, 57.36; H, 6.02; N, 11.15; (found): C, 57.30; H, 6.06; N, 11.12; FT-IR ν (cm^-1^), 3649 (COOH), 2886 (CH), 1716 (C = O), 1473 (CH = CH), 3308 (NH), 1280 (C−O), 1280 (C-N), 3586 (NH_2_). ^1^H NMR (DMSO, ppm) δ: 3.35–3.36 m, 3.85–3.86 m, 3.87–3.88 t, 6.55–6.56 t, 6.57–6.58 t, 7.43–7.44 m, 7.45–7.46 m, 7.46–7.47 m, 7.89–7.90 m, 7.92–7.93 m, (−CH−), 1.30–1.31 s, 1.09–1.10 s, (−CH_2_−), 0.58–0.59 s, 1.00–1.01 s, (−CH_3_−), 10.17 s, (−OH), 8.31 s, (−NH), 5.61 s, (−NH_2_). ^13^C NMR (DMSO, ppm) δ: 41.3 (C 1), 44.3 (C 2) (CH_3_), 46.2 (C 3), 49.8 (C 4), 50.4 (C 5), 51.5 (C 6), 56.9 (C 7), 57.3 (C 8), 59.9 (C 9), 60.5 (C 10), 66.3 (C 11) (CH_2_), 115.2 (C 12), 116.5 (C 13), 118.0 (C 14), 122.0 (C 15), 123.8 (C 16), 129.3 (C 17), 130.8 (C 18), 130.9 (C 19) (CH), 155.2 (C 20) (C-N), 166.5 (C 21), 166.7 (C 22), 166.9 (C 23), 172.0 (C 24) (C = O).

#### 3.1.2 M2: 6,6’-((oxalylbis(oxy)) bis (ethane-1,1-diyl))-bis-(3-((5-(dimethyl- carbamoyl) pyrrolidin-3-yl) thio)-4-methyl-7-oxo-1-azabicyclo [3.2.0] hept-2-ene-2-carboxylic acid)

Yield (73%); m. p. 160–161°C, insoluble in chloroform soluble in ethanol, methanol, DMSO, and distilled water. Molecular formula: C_36_H_48_N_6_O_12_S_2_ and molecular weight: 820.93 gm/mol. Elemental analysis (calculated) for C_36_H_48_N_6_O_12_S_2_: C, 52.67; H, 5.89; N, 10.24 (found) C, 52.62; H, 5.93; N, 10.28. FT-IR ν (cm^-1^), 3854 (COOH), 2947 (C−H), 1716 (C = O), 1450 (CH = CH), 1106 (C−N), 1206 (C−O), 3253 (N−H). ^1^H NMR (DMSO, ppm) δ: 3.62–3.63 m, 3.64–3.65 t, 4.16 d, 3.25 t, 3.29 t, 3.64 t, 3.34–3.44 t, 3.45–3.46 m, 3.48–3.49 m, 3.52–3.53 m, 3.55–3.56 m, 3.62–3.63 m, (−CH−), 1.34–1.35 m, 1.32–1.33 m, 1.31–1.32 m, 1.28–1.29 t, (−CH_2_−), 2.64–2.65 s, 2.62 s, 0.85–0.86 m, 0.82–0.83 m, 1.64–1.65m, 1.62–1.63 m, 1.3–1.4 m, 1.2–1.3 t, (−CH_3_−), 5.29 s, (−OH), 5.1 s, (−NH). ^13^C NMR (DMSO, ppm) δ: 26.1(C1), 30.9(C2), 34.50(C3), 36.4(C4), 37.0(C5), 39.1 (C6), 39.4 (C7), 39.6 (C8), 39.9 (C9), 40.2 (C10/11), 40.5 (C12), 40.7 (C13),44.2 (C14)(CH_3_), 52.4 (C15), 55.7 (C16), 60.8 (C17), 61.0 (C18), 68.5(C19), 70.2 (C20), 71.0 (C21) (CH_2)_, 71.7 (C22), 72.5 (C23), 73.6(C24/25), 75.9(C26),80.9(C27), 81.8(C28), 87.2 (C29), 92.4 (C30/31), 102.5(C32) (CH) 162.7(C33), 165.3(C36)(C = O).

#### 3.1.3 M3: 3-((5-(dimethylcarbamoyl)pyrrolidin-3-yl)thio)-6-(1-((2-hydro- xybenzoyl) oxy) ethyl)-4-methyl-7-oxo-1-azabicyclo[3.2.0]hept-2-ene-2-carboxylic acid

Yield (75%); m. p. 101–103°C, insoluble in chloroform and distilled water-soluble in ethanol, methanol, and DMSO. Molecular formula: C_24_H_29_N_3_O_7_S and molecular weight: 503.57gm/mol.

Elemental analysis (calculated) for C_24_H_29_N_3_O_7_S: C, 57.24; H, 5.80; N, 8.34; (found) C, 57.29; H, 5.75; N, 8.39; FT-IR ν (cm^-1^), 3885 (COOH), 2864 (C−H), 1721 (C = O), 1484 (CH = CH), 1208 (C−N), 3628 (C−O), 3185 (N−H), 3649 (O−H). ^1^H NMR (DMSO, ppm) δ: 6.88–6.87 m, 6.93–6.94 m, 6.92–6.94 m, 6.90–6.91 t, 6.91–6.92 t, 1.02–1.03, m 6.88–6.89 t, 4.76–4.77 d, 3.20–3.21 t, (−CH−), 1.00–1.02 d, (−CH_2_−), 1.5–1.56 d, 0.56–0.57 d, (−CH_3_−),7.57 s, (−NH), 9.92 s, (−OH). ^13^C NMR (DMSO, ppm) δ: 41.0(C1), 44.3(C2), 46.2(C3), 49.4(C4) (CH_3_), 50.44 (C5), 55.9(C6), 57.1(C7),59.8 (C8), 60.16(C9), 65.3 (C10), 66.3(C11) (CH_2_), 112.9(C12), 115.7(C13), 117.0(C14), 119.1(C15), 123.8(C16), 129.3(C17), 130.2(C18), 135.5(C19)(CH), 159.2 (C20) (C-N), 161.0(C21), 165.4(C22), 171.7(C23), 172.3(C24) (C = O).

#### 3.1.4 M4: 6,6’-((succinylbis(oxy))bis(ethane-1,1-diyl))bis(3-((5-(dimethylcar bamoyl) pyrrolidin-3-yl)thio)-4-methyl-7-oxo-1-azabicyclo[3.2.0]hept-2-ene-2-carboxylic acid)

Yield (7079.88%), m.p 120–122°C insoluble in chloroform and distilled water-soluble in ethanol, methanol, and DMSO. Molecular formula: C_38_H_52_N_6_O_12_S_2_ and molecular weight: 848.9 gm/mol.

Elemental analysis (calculated) for C_38_H_52_N_6_O_12_S_2_: C, 53.76; H, 6.17; N, 9.90; (found): C, 53.70; H, 6.21; N, 9.85; FT-IR ν(cm^-1^), 3860 (COOH), 2883 (C−H), 1718 (C = O), 1653 (CH = CH), 1208 (C−N), 1023(C−O), 3149 (N−H). ^1^H NMR (DMSO, ppm) δ: 3.16–3.7 d, 3.38–3.39 t, 3.41–3.42 t, 3.42–3.43 t, 3.44–3.45 t, (−CH−), 1.00–1.01 d, (−CH_2_−), 2.50–2.51 d, (−CH_3_−), 5.96 s, (−OH), 7.32 s, (−NH). ^13^C NMR (DMSO, ppm) δ: 41.29(C 1), 44.3(C 2), 46.2(C 3), 49.8(C 4) (CH_3_), 50.4 (C 5), 51.5 (C 6), 56.9 (C 7), 57.3 (C 8), 59.9 (C 9), 60.5(C 10), 66.3 (C 11) (CH_2_), 115.2 (C 12), 116.5(C 13), 118.0 (C 14), 122.0 (C 15), 123.8 (C 16), 129.3 (C 17), 130.8 (C 18), 130.9 (C 19)(CH), 155.2 (C 20) (C-N)), 166.5(C 21), 166.7 (C 22), 166.9 (C 23), 172.0 (C 24) (C = O).

#### 3.1.5 M5:6,6’-(((2-aminopentanedioyl)-bis-(oxy))-bis-(ethane-1,1-diyl))-bis-(3-((5-(dimethyl carbamoyl) pyrrolidin-3-yl)thio)-4-methyl-7-oxo-1-azabicyclo [3.2.0] hept-2-ene-2-carboxylic acid)

Yield (73%), m.p 133–134°C insoluble in chloroform but soluble in ethanol, methanol, DMSO, and distilled water. Molecular formula: C_39_H_55_N_7_O_12_S_2_ and molecular weight: 878.03 gm/mol.

Elemental analysis (calculated) for C_39_H_55_N_7_O_12_S_2_: C, 53.35; H, 6.31; N, 11.17; (found): C, 53.40; H, 6.35; N, 11.12; FT-IR ν (cm^-1^), 3869 (COOH), 2920 (CH), 1711 (C = O), 1638 (CH = CH), 1306 (C−N), 1086 (C−O), 3125 (NH), 3329 (NH_2_). ^1^H NMR (DMSO, ppm) δ: 3.12–3.13 t, 3.57–3.58 t, 3.62–3.63 m, 3.91–3.92 t, 3.40–3.41 m, (−CH−), 1.05–1.06 d, (−CH_2_−), 2.51–2.52 d, 2.32–2.33 d, (−CH_3_−), 11.02 s, (−OH), 7.82 s, (NH). ^13^C NMR (DMSO, ppm) δ: 39.1(C1), 39.4 (C2), 39.6 (C3), 39.9 (C4), 40.2 (C5/6), 40.5(C7), 40.7(C8) (CH_3_), 61.7(C9), 65.2 (C10), 70.4 (C11/12), 74.9 (C13), 79.0 (C14), 79.4 (C15), 79.8 (C16), 81.2 (C17), 85.9 (C18), 92.2 (C19/20) (CH_2_), 145.4 (C21), 147.0 (C22)(C), 151.2 (C23), 157.7 (C24/25)(C-N), 160.2 (C26), 160.9 (C27/28), 165.2 (C29), 167.3 (C30), 169.9 (C31), 171.1 (C32/33), 173.5(C34), 175.1(C35/36), 176.8(C37), 177.2(C38/39) (C = O).

#### 3.1.6 M6:3-((5-(dimethylcarbamoyl)pyrrolidin-3-yl)thio)-4-methyl-7-oxo-6-(1-((3,4,5trihydroxy-benzoyl) oxy)ethyl)-1-azabicyclo[3.2.0]hept-2-ene-2-carboxylic acid

Yield (72%); m.p. 147–149°C insoluble in chloroform but soluble in ethanol, methanol, DMSO, and distilled water. Molecular formula: C_24_H_29_N_3_O_9_S and molecular weight: 535.57 gm/mol.

Elemental analysis (calculated) for C_24_H_29_N_3_O_9_S: C, 53.82; H, 5.46; N, 7.85; (found); C, 53.78; H, 5.51; N, 7.81; FT-IR ν (cm-^1^), 3743 (COOH), 2919 (C−H), 1711 (C = O), 1637 (CH = CH), 1307 (C−N), 1087 (C−O), 3616 (O−H), 3190 (N−H). ^1^H NMR (DMSO, ppm) δ: 3.33–3.34 d, 4.13–4.14 m,4.17–4.18 m, (−CH−), 1.28–1.29 d,1.33–1.34 m,1.37–1.39 m, (−CH_2_−) 0.88–0.89 m, 1.23–1.24 m, (−CH_3_−),4.18 s, (−OH), 7.73 s, (−NH). ^13^C NMR (DMSO, ppm) δ: 14.2(C1), 14.3(C2), 21.8(C3), 34.1(C4), 40.5 (C5)(CH_3_), 59.9 (C6)(CH_2_), 108.4 (C7), 108.5 (C8), 108.7(C9), 119.6 (C10), 120.4 (C11), 138.3 (C12) (CH), 145.3 (C13), 145.5 (C14), 149.9 (C15)(C), 151.3 (C16), 153.4 (C17), 155.9 (C18) (C-N), 158.7 (C19), 161.2 (C20), 163.5 (C21), 165.8 (C22/23), 167.4 (C24) (C = O).

#### 3.1.7 M7:6,6’-(((2,3-dihydroxysuccinyl)bis(oxy))bis(ethane-1,1-diyl))bis(3-((5-(dimethy-lcarbam-oyl)pyrrolidin-3-yl)thio)-4-methyl-7-oxo-1-azabicyclo [3.2.0]hept-2-ene-2-carboxylic acid)

Yield: (75%), m.p. 108–110°C insoluble in chloroform but soluble in ethanol, methanol, DMSO, and distilled water. Molecular formula: CH_52_N_6_O_14_S_2_ and Molecular weight: 880.98 gm/mol.

Elemental analysis (calculated) for C_38_H_52_N_6_O_14_S_2_: C, 51.81; H, 5.95; N, 9.54; (found): C, 51.85; H, 5.92; N, 9.51; FT-IR ν (cm-^1^), 3853 (COOH), 2991 (C−H), 1734 (C = O), 1653 (CH = CH), 1266 (C−N), 1653 (C−O), 3628 (O−H), 3170 (N−H). ^1^H NMR (DMSO, ppm) δ: 3.16–3.17 t, 3.52–3.53 t, 3.62–3.63 m, 3.94–3.95 t, 3.97–3.98 t,3.41–3.42 m, (−CH−), 1.2–1.3 d, (−CH_2_−), 2.49–2.50 d, 2.53–2.54 d, (−CH_3_−), 8.87 s, (−NH), 14.41 s, (−OH). ^13^C NMR (DMSO, ppm) δ: 19.0 (C1), 39.3 (C2), 39.5 (C3), 39.7 (C4), 39.9 (C5), 40.2 (C6), 40.6 (C7)(CH3), 56.5 (C8), 75.2 (C9), 77.4 (C10), 79.9 (C11), 85.2(C12), 88.9 (C13), 92.5 (C14), 95.7 (C15), 99.2 (C16) (CH2), 104.2 (C17), 107.1(C18), 111.1 (C19), 112.5 (C20), 120.3 (C21), 122.1 (C22), 124.5 (C23/24), 129.5 (C25) (CH), 130.2 (C26), 130.9 (C27/28)(CH), 138.9 (C29), 157.7 (C30/31/32), 153.4 (C33), 155.2 (C34/35), 158.7 (C36) (C-N), 159.1(C37), 160.3(C38) (C = O).

#### 3.1.8 M8: 6,6’-((phthaloylbis(oxy))bis(ethane-1,1-diyl))bis(3-((5-(dimethyl carbamoyl) pyrrolidin-3-yl)thio)-4-methyl-7-oxo-1-azabicyclo[3.2.0]hept-2-ene-2-carboxylic acid)

Yield: (79%); m. p 145–147°C insoluble in chloroform but soluble in ethanol, methanol, DMSO, and distilled water. Molecular formula: C_42_H_52_N_6_O_12_S_2_ and molecular weight: 897.03 gm/mol. Elemental Analysis for C_42_H_52_N_6_O_12_S_2_ (calculated), C, 56.24; H, 5.84; N, 9.37; found: C, 56.22; H, 5.87; N, 9.31.

FT-IR ν (cm-1), 3853 (COOH), 2990 (C−H), 1716 (C = O), 1473 (CH = CH), 1166 (C−N), 1372 (C−O), 3525 (N−H). 1H NMR (DMSO, ppm) δ: 3.16–3.17 t, 3.52–3.53 t, 3.62–3.63 m, 3.94–3.95 t, 3.41–3.42 m, (−CH−), 1.3–1.4 d, (−CH2−), 2.47–2.48 d, 2.52–2.53 d, (−CH3−), 14.41 s, (−OH), 8.87 s, (−NH). 13C NMR (DMSO, ppm) δ: 40.3(C1), 41.0 (C2), 44.3 (C3), 46.2 (C4), 49.4(C5)(CH3), 50.2 (C6), 52.3 (C7), 57.1 (C8), 59.8 (C9), 60.5 (C10), 61.1 (C11), 66.3 (C12)(CH2), 128.1 (C13), 129.3(C14), 130.7 (C15), 131.2 (C16), 131.3 (C17), 131.4 (C18), 131.5 (C19), 131.9 (C20), 132.0 (C21), 132.3 (C22), 132.4 (C23), 132.7 (C24)(CH), 159.2 (C25), 166.7 (C26/27), 166.8 (C28), 166.8 (C29), 166.9 (30), 167.4 (C31), 167.5, (C32/33), 167.8 (C34), 167.9 (C35/36/37), 168.0 (C38), 168.6 (C39,40), 172.0 (C41),172.3 (C42) ((C = O).

### 3.2 Biological evaluation

#### 3.2.1. Antibacterial activity

The results of antibacterial studies were determined and tabulated in Table 1.

**Table 1:** The results of antibacterial studies were determined and tabulated against gram-positive and gram-negative bacterial strains.

Description	Concentration (μg.ml^-1^)	Gram positive bacterial strains				Gram negative bacterial strains		
		*Bacillus megatarim*	*Bacillus subtilis*	*Staphylococcus aureus*	*Micrococcus luteus*	*Stenotrophomonas maltophilia*	*Serratia marcescens*	*Escherichia coli*
**M_1_**	40 μg.ml^-1^	19 ±0.81	13±0.81	11±0.81	20±0.81	18 ±0.81	16±0.81	12±0.81
	60 μg.ml^-1^	20± 0.81	15±0.81	14±0.81	21±0.471	19 ±0.47	19±0.81	15±0.81
	80 μg.ml^-1^	22 ±0.81	18±0.81	18±0.81	23±0.81	21 ±0.81	20±0.81	19±1.63
**M_2_**	40 μg.ml^-1^	27±0.81	26±0.81	30±0.81	32±0.81	24 ±0.47	19±1.24	19±1.24
	60 μg.ml^-1^	29±0.81	29±0.81	33±0.81	25±0.81	29 ±0.81	22±0.25	22±0.81
	80 μg.ml^-1^	33± 0.47	34±0.81	35±0.81	32±0.81	33 ±0.81	29±0.81	28±0.81
**M_3_**	40 μg.ml^-1^	28 ±0.81	15±0.81	25±0.81	24±0.81	22 ±0.47	27±0.81	20±1.63
	60 μg.ml^-1^	30 ±0.81	19±0.81	27±0.81	26±0.471	27 ±0.81	25±0.81	22±1.24
	80 μg.ml^-1^	32± 0.81	20±0.81	30±0.471	30±0.81	30 ±0.81	36±2.05	26±0.81
**M_4_**	40 μg.ml^-1^	18± 0.81	23±0.81	22±0.81	23±0.81	21 ±0.81	23±1.24	18±0.81
	60 μg.ml^-1^	19± 0.81	24±0.81	27±0.81	28±0.81	28 ±0.81	25±0.81	20±1.24
	80 μg.ml^-1^	20 ±0.81	31±0.81	32±0.81	31±0.81	30 ±0.81	31±0.81	24±1.69
**M_5_**	40 μg.ml^-1^	25 ±0.81	20±0.81	21±0.81	24±0.81	20 ±0.81	21±0.81	21±0.81
	60 μg.ml^-1^	14± 0.81	24±0.471	25±0.81	28±0.471	26 ±0.81	24±1.24	21±1.24
	80 μg.ml^-1^	27± 0.471	30±0.81	27±0.471	13±0.81	25 ±0.471	25±1.24	30±1.41
**M_6_**	40 μg.ml^-1^	22± 0.471	21±0.81	20±0.81	25±0.471	18 ±0.81	19±0.81	20±0.81
	60 μg.ml^-1^	18± 0.81	20±0.96	19±0.471	21±0.471	22 ±0.81	21±0.81	22±0.47
	80 μg.ml^-1^	21± 0.81	25±0.81	21±0.89	16±0.471	21 ±0.81	22±0.81	25±0.81
**M_7_**	40 μg.ml^-1^	26± 0.471	16±0.81	23±0.471	-	14 ±0.81	18±0.81	19±1.24
	60 μg.ml^-1^	27±0.471	18±0.81	20±0.81	-	24 ±0.471	20±0.81	19±2.05
	80 μg.ml^-1^	29± 0.471	19±0.81	28±0.471	-	28 ±0.81	24±0.471	27±1.24
**M_8_**	40 μg.ml^-1^	27± 0.81	20±0.81	26±0.471	21±0.81	23 ±0.471	26±0.471	16±1.63
	60 μg.ml^-1^	30± 0.81	21±0.81	25±0.81	25±0.81	28 ±0.81	26±2.05	18±0.81
	80 μg.ml^-1^	32± 0.471	23±0.47	28±0.81	30±0.471	30 ±0.94	27±0.81	20±0.81
**Positive Control**	40 μg.ml^-1^	20±0.81	15±0.47	12±0.81	20±0.81	19±0.81	19±0.81	18±0.81
	60 μg.ml^-1^	21±0.47	18±0.81	13±0.81	22±0.81	20 ±0.81	20±0.81	21±0.81
	80 μg.ml^-1^	24±0.81	20±0.81	17±1.24	25±0.47	22±0.47	23±0.94	27±1.63

Escherichia coli ATCC-BAA 2471™, Stenotrophomonas maltophilia ATTC 13637™, Bacillus megatarium ATTC 14581™, Micrococcus luteus ATTC 10240b™, Bacillus subtilis ATTC 6051™, Staphylococcus aureus ATTC 700699™, and Serratia marcescens ATTC 14756™, 5% DMS used as negative control

#### 3.2.2 Enzyme inhibition activity

3.2.2.1 Urease Inhibition and alpha-amylase inhibition assays (Table 2).

**Table 2:** The results of urease inhibition assay of meropenem and its derivatives.

Description	Urease inhibition assay						α-amylase inhibition assay					
	y (Abs) 1mg.ml^-1^	b	a	v (ml)	m (gm)	Amount (mg.TU.Eq.gm^-1^)	Abs	b	a	v (ml)	m (gm)	Amount (mg.Acar Eq.gm^-1^)
**Meropenem**	0.8531	-0.371	2.428	0.03	0.00003	74.147±14.62	0.184	0.0969	0.146	0.03	0.00003	0.272±0.031
**M_1_**	0.188	-0.371	2.428	0.03	0.00003	441.036±11.76	0.179	0.0969	0.146	0.03	0.00003	10.17±2.06
**M_2_**	0.0255	-0.371	2.428	0.03	0.00003	683.329±6.51	0.37	0.0969	0.146	0.03	0.00003	07.351±0.160
**M_3_**	0.1155	-0.371	2.428	0.03	0.00003	536.148±7.152	0.226	0.0969	0.146	0.03	0.00003	24.32±4.76
**M_4_**	0.616	-0.371	2.428	0.03	0.00003	139.809±19.65	0.398	0.0969	0.146	0.03	0.00003	09.135±0.466
**M_5_**	0.6905	-0.371	2.428	0.03	0.00003	119.964±53.65	0.207	0.0969	0.146	0.03	0.00003	13.35±1.55
**M_6_**	0.6515	-0.371	2.428	0.03	0.00003	31.729±31.72	0.423	0.0969	0.146	0.03	0.00003	18.96±2.20
**M_7_**	0.085	-0.371	2.428	0.03	0.00003	582.166±15.53	0.446	0.0969	0.146	0.03	0.00003	15.86±2.422
**M_8_**	0.210	-0.371	2.428	0.03	0.00003	416.439±38.04	0.17	0.0969	0.146	0.03	0.00003	9.032±0.329

M_1-8_ represents synthesized ester derivatives, and all values are mean ± SD of triplicate observations, mg.TU.Eq.gm^-1^ means milligrams thiourea equivalent per gram of sample; mg Acar Eq.gm^-^ means milligrams acarbose equivalent per gram of sample. Amount of Trolox equivalent and Acarbose equivalent per gram of sample M_1-8_ and meropenem a parent drug was determined using straight line equation (y = bx + a)

#### 3.2.3 Antioxidant activity

The free radical scavenging potential of 3-((5- (dimethylcarbamoyl)pyrrolidin-3-yl) thio)-6- (1-hydroxyethyl)-4-methyl-7-oxo-1-azabicyclo[3.2.0]heptane-2-carboxylic acid and its derivatives was determined and results presented in Table 3.

**Table 3:** DPPH radical scavenging activity of meropenem, its derivatives (M_1_-M_8_) and ascorbic acid.

Description	Conc.(mg/ml)	% Inhibition ± S. D
**M_1_**	0.5	-
**M_2_**	0.5	52 ± 0.47
**M_3_**	0.5	-
**M_4_**	0.5	52.77 ± 0.47
**M_5_**	0.5	-
**M_6_**	0.5	90.91 ± 0.45
**M_7_**	0.5	-
**M_8_**	0.5	81.48 ± 0.61
**Meropenem**	0.5	58.68 ± 0.41
**Ascorbic Acid**	0.5	96.00 ± 0.23%

M(std) represents the parent drug meropenem, M(1–8) represents synthesized ester derivatives, all values are mean ± SD of triplicate observations, and (-) indicates no activity.

### 3.3 Molecular docking and ADME pharmacokinetics properties

Tables 4 and 5 show the outcomes of in-silico molecular docking and ADME pharmacokinetics parameters, respectively. The *In-silico* molecular docking study included 3-((5- (dimethylcarbamoyl) pyrrolidin-3-yl) thio)-6- (1-hydroxyethyl)-4-methyl-7-oxo-1-azabicyclo [3.2.0] heptane-2-carboxylic acid and its synthesized derivatives against two clinically important enzymes i.e., urease and alpha-amylase.

**Table 4:** The results of *In-silico* molecular docking of the parent molecule and its structural analogues.

Compound	AMYLASE (5E0F)			UREASE (4H9M)		
	Binding Energy	Ref RMSD	Inhibition Constant(ki)	Binding Energy	Ref RMSD	Inhibition Constant (ki)
**M**_**1**_	-5.10	21.59	182.07 μM	-3.87	66.91	1.46 mM
**M**_**2**_	-5.45	23.32	101.77 μM	-6.12	64.43	32.53 μM
**M**_**3**_	-5.92	22.16	45.86 μM	-5.43	65.33	104.61 μM
**M**_**4**_	-9.44	21.25	120.27 nM	-13.14	61.84	233.52 pM
**M**_**5**_	-6.03	24.01	38.04 μM	-5.94	65.00	44.45 μM
**M**_**6**_	-7.05	20.91	6.81 μM	-7.42	65.22	3.67 μM
**M**_**7**_	-7.32	26.62	4.30 μM	-8.19	63.58	988.76 nM
**M**_**8**_	-9.10	22.76	214.79 nM	-	-	-
**Positive control Drug**	-6.39	21.87	20.71 μM	-8.37	66.43	731.21 nM
**Co-crystalline ligand**	-4.43	23.36	562.43 μM	-3.43	59.00	3.04 mM

**Table 5:** Predicted ADME properties of derivatives of 3-((5-(dimethylcarbamoyl)pyrrolidin-3-yl)thio)-6-(1-hydroxyethyl)-4-methyl-7-oxo-1-azabicyclo[3.2.0]heptane-2-carboxylic acid.

Compound	Popular surface area	GI absorption	BBB permeant	Lipinsi violations	Ghose violatios	Bioavailability Score	% absorption
**M_1_**	164.7	Low	No	0	1	0.17	51.2
**M_2_**	268.08	Low	No	2	4	0.17	16.5
**M_3_**	157.51	Low	No	2	4	0.17	54.6
**M_4_**	292.12	Low	No	2	3	0.17	8.2
**M_5_**	321.28	Low	No	2	3	0.17	-1.8
**M_6_**	197.73	Low	No	2	1	0.17	40.7
**M_7_**	318.42	Low	No	2	3	0.17	-0.8
**M_8_**	286.43	Low	No	2	3	0.17	10.2

### 3.4 ADME pharmacokinetics studies

The predicted ADME parameters of all the tested compounds were determined and tabulated in Table 5.

## 4. Discussion

**Fig 3:**
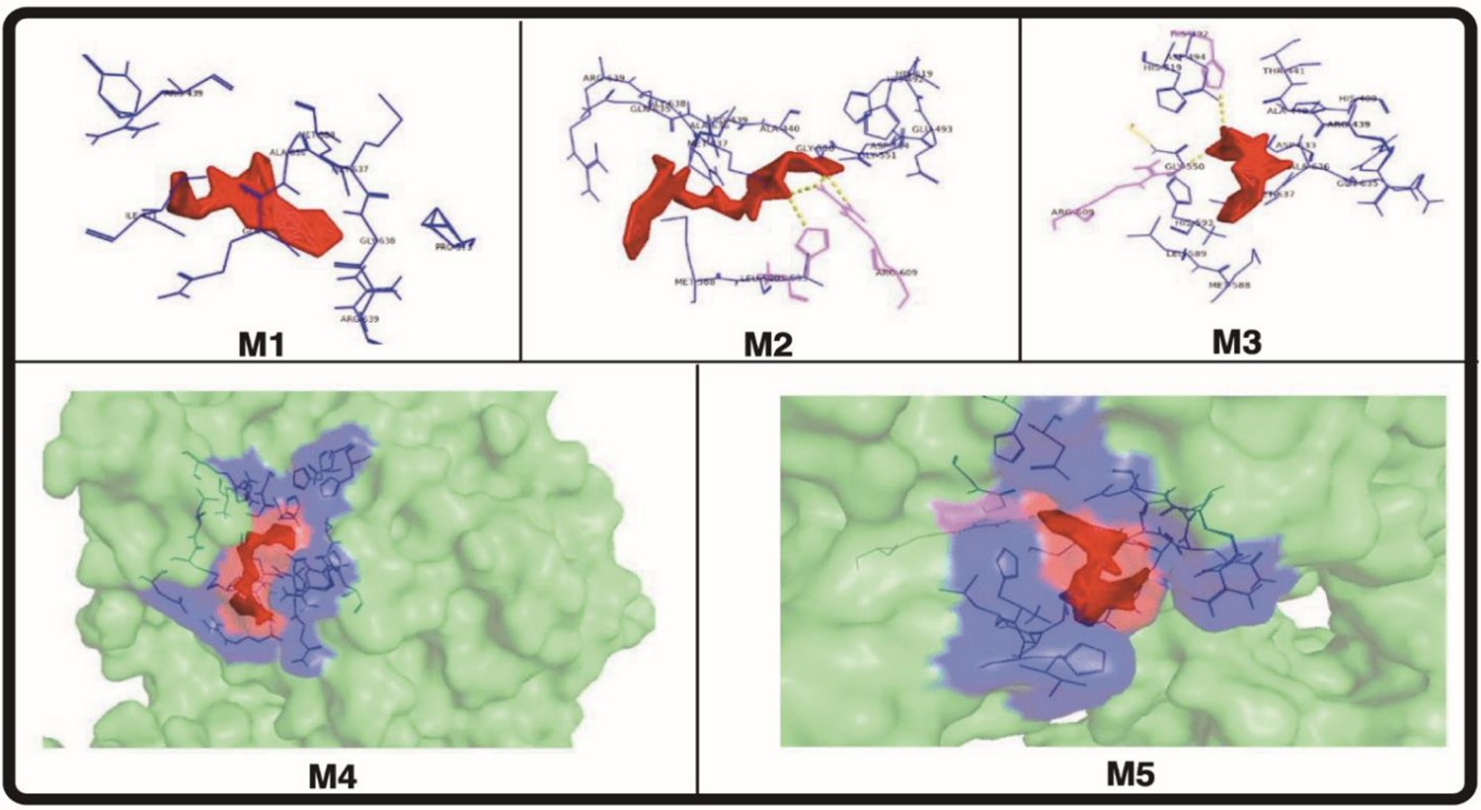
*In-silico* molecular docking of ligands with urease (4H9M).

**Fig 4:**
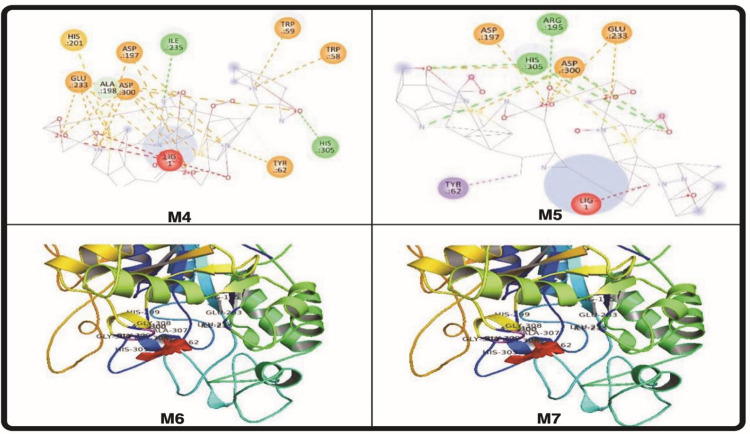
*In-silico* molecular docking of ligand with alpha amylase (5E0F).

**Fig 5:**
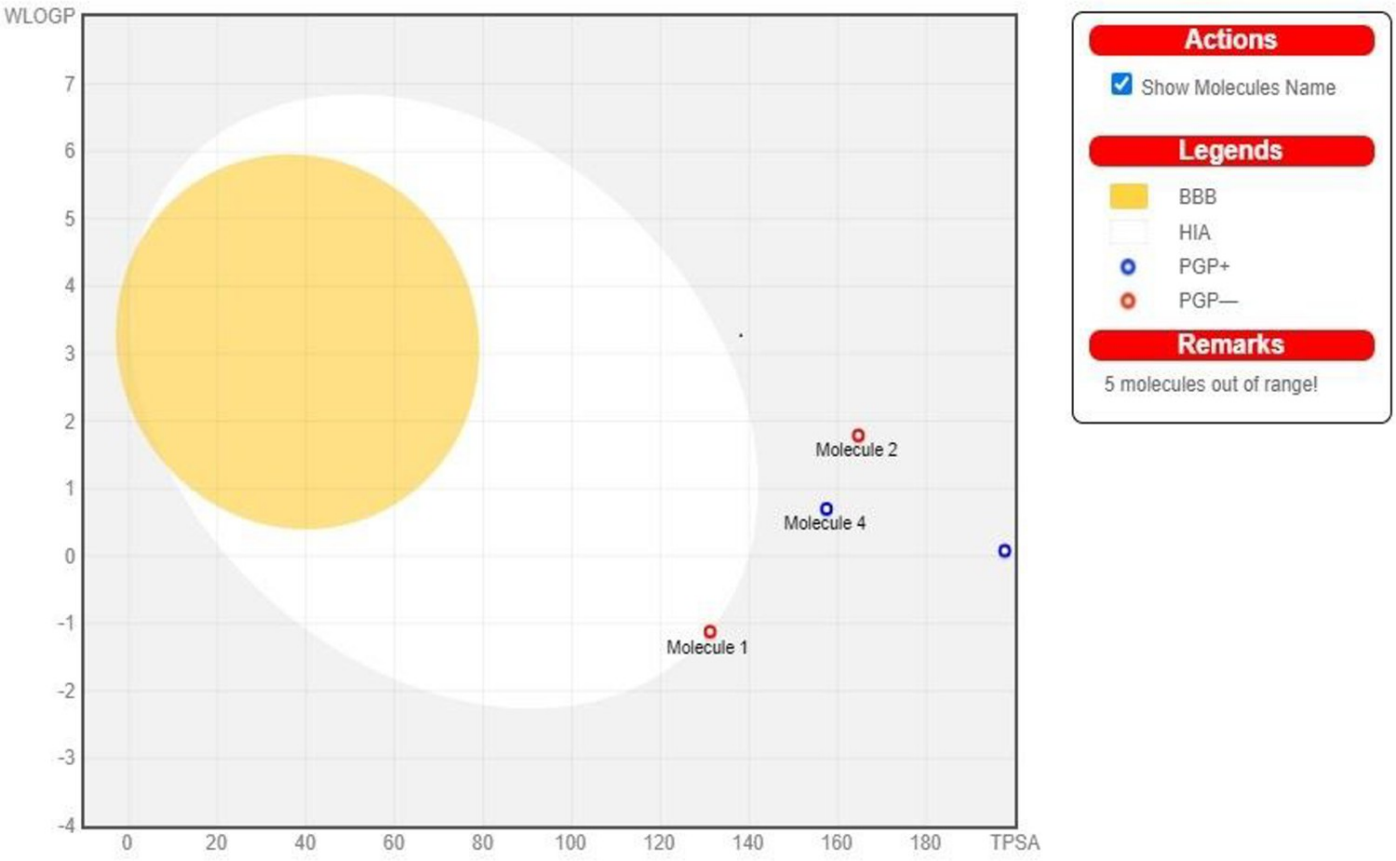
Egg plot "a correlation between antibacterial activity and ADME parameter.

In this investigation, Fischer esterification was used to create meropenem ester derivatives. The enhanced PBP and potent action of meropenem were caused by the presence of a pyrrolidine ring in the drug’s structure. Penicillin-binding proteins (PBPs), which are important in the manufacture of bacterial cell walls, attach to it covalently, and when they are inhibited, cell death results [22]. Compared to gram positive bacterial strains, it is more effective against gram negative bacterial strains. Except for PBP3, every PBP in *Staphylococcus aureus* had a greater affinity for meropenem [23]. Meropenem antimicrobial resistance was primarily caused by changes in PBPs, plasmid-mediated beta-lactamases, and altered bacterial membrane permeability [18]. An exhaustive study of the literature found that aromatic esters have improved anti-microbial and antioxidant effects [24]. Against the gram-negative bacterial strain *Bacillus megatarium*, M_2_, M_3_, M_7_, and M_8_ had zones of inhibition that were larger than those of meropenem parent drug. When tested against *Bacillus subtilis*, the compounds M_2_, M_4_, M_6_, and M_8_ also showed strong antibacterial properties. Because of PBP3, *Staphylococcus aureus* displayed the lowest binding affinity to M (std) drug, although compounds M_2_, M_3_, M_4_, M_5_, M_7_, and M_8_ exhibited a greater zone of inhibition (Table 1). As indicated in Table 1, the M_7_ had little action against *Micrococcus luteus*, but the M_2_, M_4_, and M_8_ compounds displayed high activity. An increase in lipophilicity, a significant factor in the antibacterial activity that improves the permeability of ester derivatives into the lipid membrane and inhibits bacterial growth, may be the source of an increase in antibacterial activity. According to Table 1, M_2_ and M_3_ demonstrated enhanced activity against *Escherichia coli*, whereas M_3_, M_4_, and M_8_ showed improved activity against *Serratia marcescens*. According to a review of the literature, clarithromycin’s ability to treat peptic ulcers brought on by Helicobacter pylori is waning. Several studies also showed that blocking the urease enzyme is directly related to bacterial death. Few investigations have found that recently synthesized antibacterial also had anti-diabetic properties. To investigate anti-urease and alpha-amylase inhibition, both enzymes were chosen. When compared to thiourea and the parent drug meropenem, in vitro enzyme inhibition of M_1_-M_8_ against jack bean urease exhibited much higher activity. In Table 2, where acarbose was utilized as a positive control, the findings of the -amylase inhibition experiment was not encouraging. Similarly, neither the original drug molecule nor any of its synthesized derivatives, with the exception of M_2_, M_4_, M_6_, and M_8_, showed any promising antioxidant potential (Table 3). Docking analysis was conducted, as shown in Table 4, to estimate the potential mode of interaction between the substantially active and least active chemical and the target protein. The derivative M_4_ showed high action against urease and alpha-amylase, according to computational investigations as indicated in Table 4. Compound ADMET characteristics are crucial for drug development and design. All meropenem derivatives had strong anti-urease action, however compounds M_4_ and M_7_ were found to be more powerful than thiourea, which was utilized as a positive control. When linked with ALA636, ARG439, and HIS539 at catalytic pocket sites, the compounds M_4_ and M_7_ demonstrated robust hydrogen bonding. A salt bridge involving the amino acids ARG439 and ARG639 was seen in M7. ILE411, ALA440, and ALA636 demonstrated hydrophobic interaction with M_1_, M_3_, and M_6_. Fig 3 illustrates the compounds M_2_ and M_5_ forming a salt bridge with HIS593 and ARG609. Additionally, docking tests on meropenem derivatives were carried out to look at their affinities for binding to amylase. In the catalytic pocket, the hydrophobic amino acid on the active site appears to be essential for ligand binding. Meropenem and its synthetic analogues did not have attractive docking results, although some of them had hydrophobic interaction, including TRP59, TYR62, ILE162, ALA198, LYS200, and HIS201, as well as hydrogen bonding in HIS195, ILE235, HIS305, and GLY309. Pi-stacking was seen with the compounds M_4_ and M_8_ with TYR62, HIS201, and HIS301. Fig 4 depicts a salt bridge between M_5_ and M_8_ and ARG195, HIS299, and HIS305. To evaluate the pharmacokinetic and safety profile of the synthesized molecules M_1_-M_8_, ADMET properties were estimated. Each compound has noteworthy physicochemical characteristics. Meropenem derivatives in general exhibited no Lipinski violations. The synthesized analogs of meropenem are all shown in Table 5 above, and according to the antibacterial results and ADMET parameters findings, none of them crossed the blood-brain barrier and all compounds have low gastrointestinal absorption, demonstrating that derivatives are good for topical use and can be further utilized for investigation by in vivo studies for better contributions to science and medicine (Fig 5).

## 5. Conclusion

We synthesized many meropenem ester compounds and investigated their antibacterial, antioxidant, and enzyme-inhibitory properties. M_2_ and M_3_ were discovered to have stronger antibacterial effects than the parent medication. The anti-urease potential of M_1_, M_2_, M_3_, and M_6_ was also notable and found to be in good accord with their molecular docking results, offering important insight into a potential mechanism of action. Thus, it is concluded that there is a large area for more study and compound optimization, which may result in the creation of some potential antibacterial drugs to combat bacterial resistance in a variety of bacterial illnesses.

## Supporting information

S1 File **Part A.** Antibacterial activity (Figs 1–7 of S1 File) of synthesized derivatives M_1_-M_8_ and meropenem, **Part B. FTIR** spectrum (Figs 8–16 of S1 File) of synthesized derivatives M_1_-M_8_ and meropenem, **Part C.** ^1^Proton and ^13^carbon NMR (Figs 17–33 of S1 File) of synthesized derivatives M_1_-M_8_ and meropenem.
(DOCX)
