# Supplementary material for: Synthesis, antimicrobial and molecular docking study of structural analogues of 3-((5-(dimethylcarbamoyl)pyrrolidin-3-yl)thio)-6-(1-hydroxyethyl)-4-methyl-7-oxo-1-azabicyclo[3.2.0]heptane-2-carboxylic acid
Source: PLoS One. 2022 Dec 27;17(12):e0278684. doi: 10.1371/journal.pone.0278684 (PMC9794083; doi:10.1371/journal.pone.0278684)
Supplement: S1 File — Part A. Antibacterial activity (Figs 1–7 of S1 File) of synthesized derivatives M1-M8 and meropenem, Part B. FTIR spectrum (Figs 8–16 of S1 File) of synthesized derivatives M1-M8 and meropenem, Part C. 1Proton and 13carbon NMR (Figs 17–33 of S1 File) of synthesized derivatives M1-M8 and meropenem. (DOCX) [file pone.0278684.s001.docx]

**MEASUREMENT OF ZONE OF INHIBITION AGAINST DIFFERENT BACTERIA**

**Part A.**


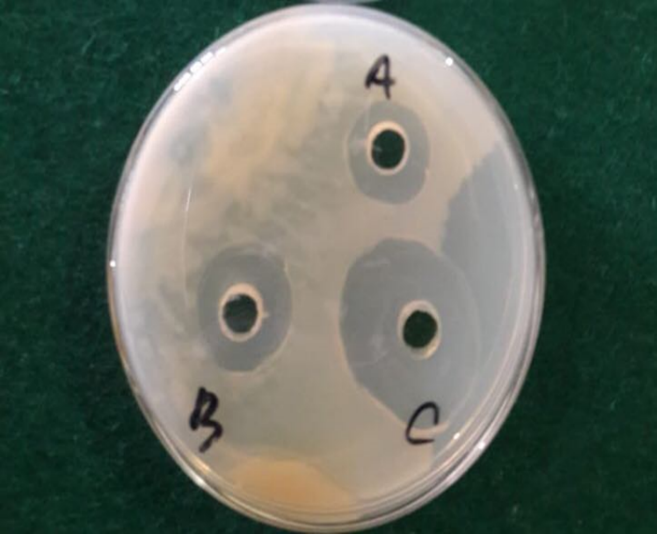


Figure 1. Meropenem (Standard) against Micrococcus luteus


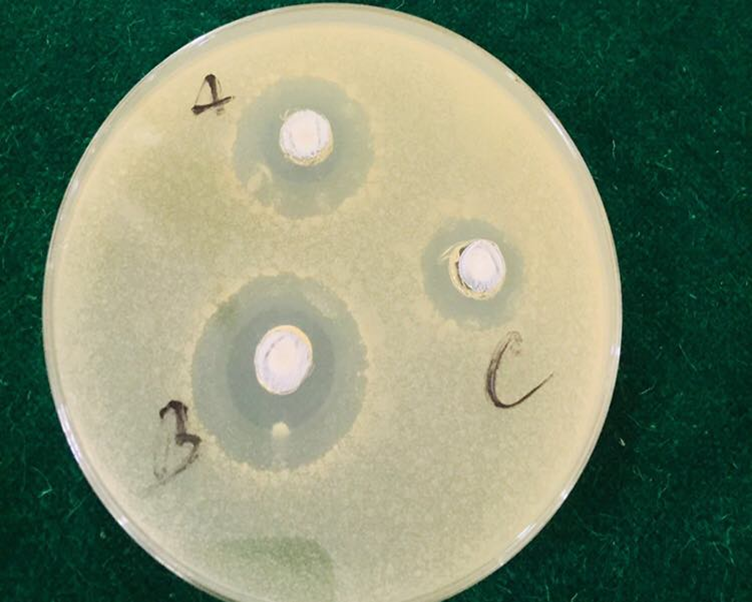


Figure 2. M5 against Micrococcus luteus


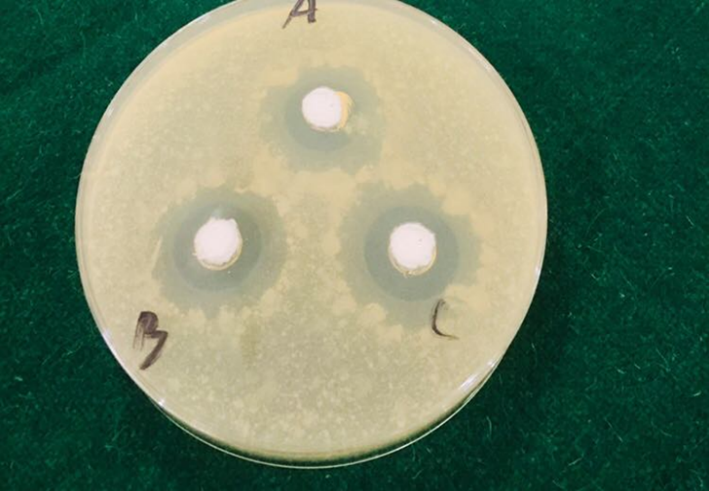


Figure 3. M8 against Staphylococcus aureus


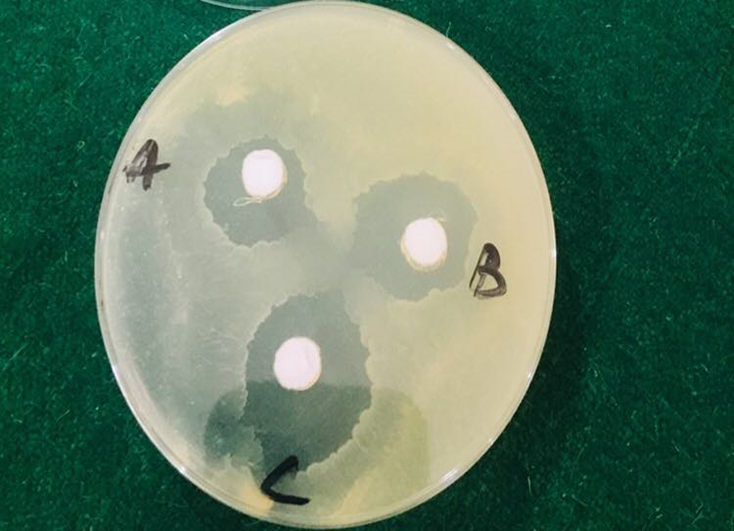


Figure 4. M2 against Serratia marcescens


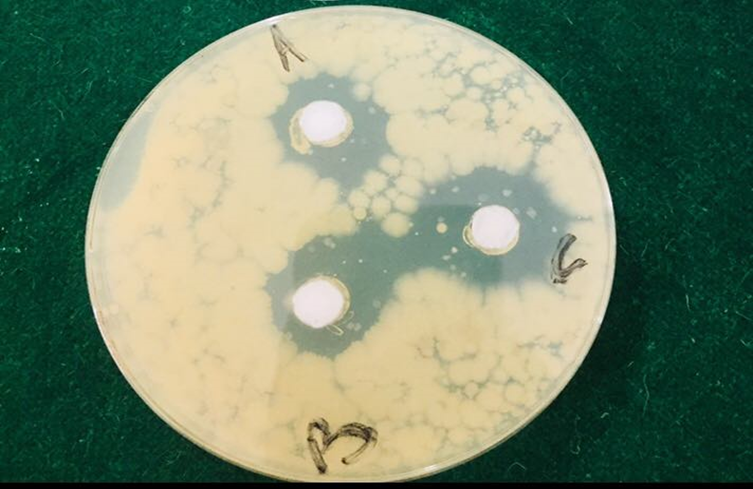


Figure 5. M2 against E.coli


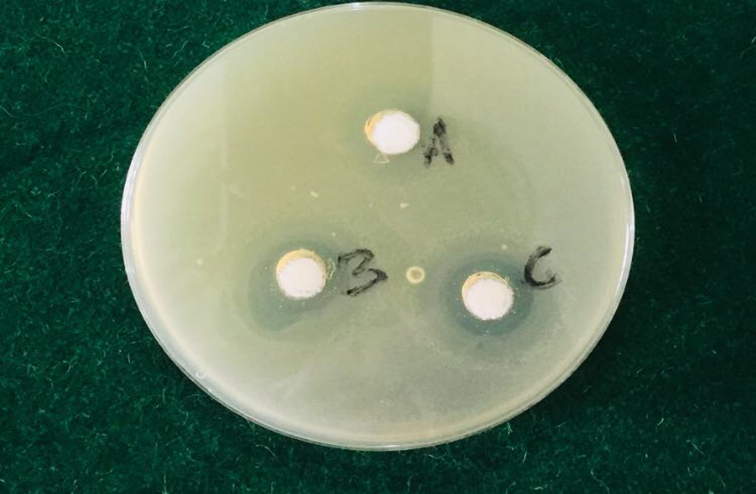


Figure 6. M4 against Stenotrophomonas maltophilia


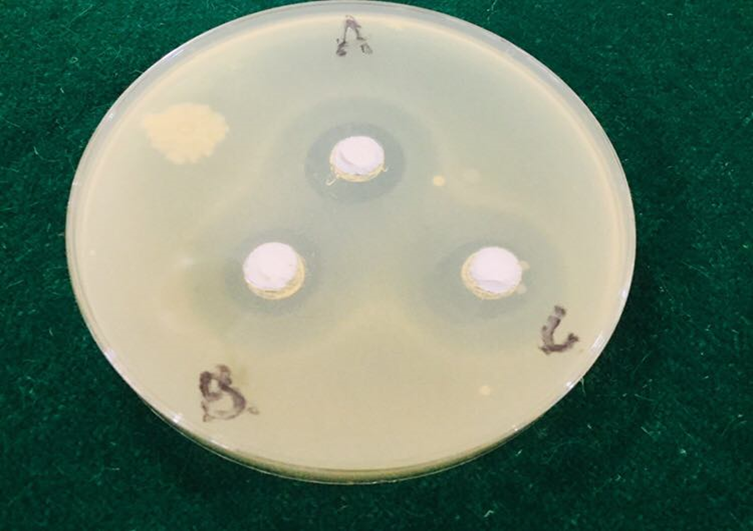


Figure 7. M7 against Bacillus megatarium

**FTIR (part B)**

**M (std)**


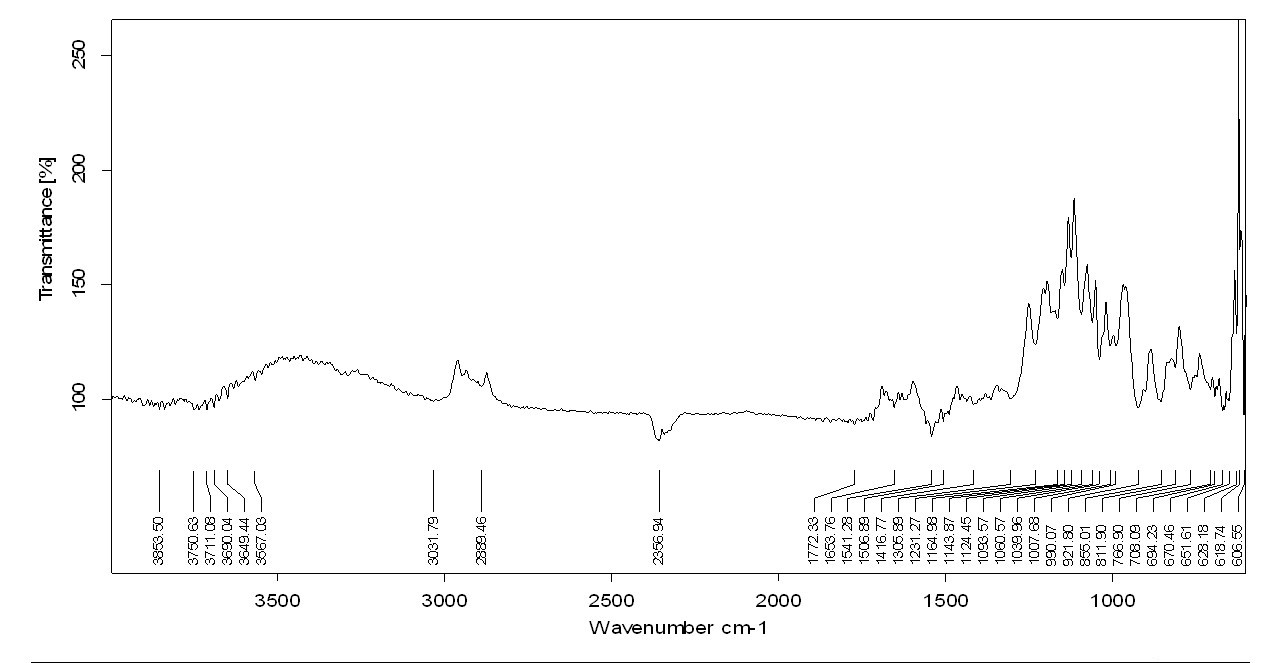


Figure 8. FTIR spectrum of Meropenem


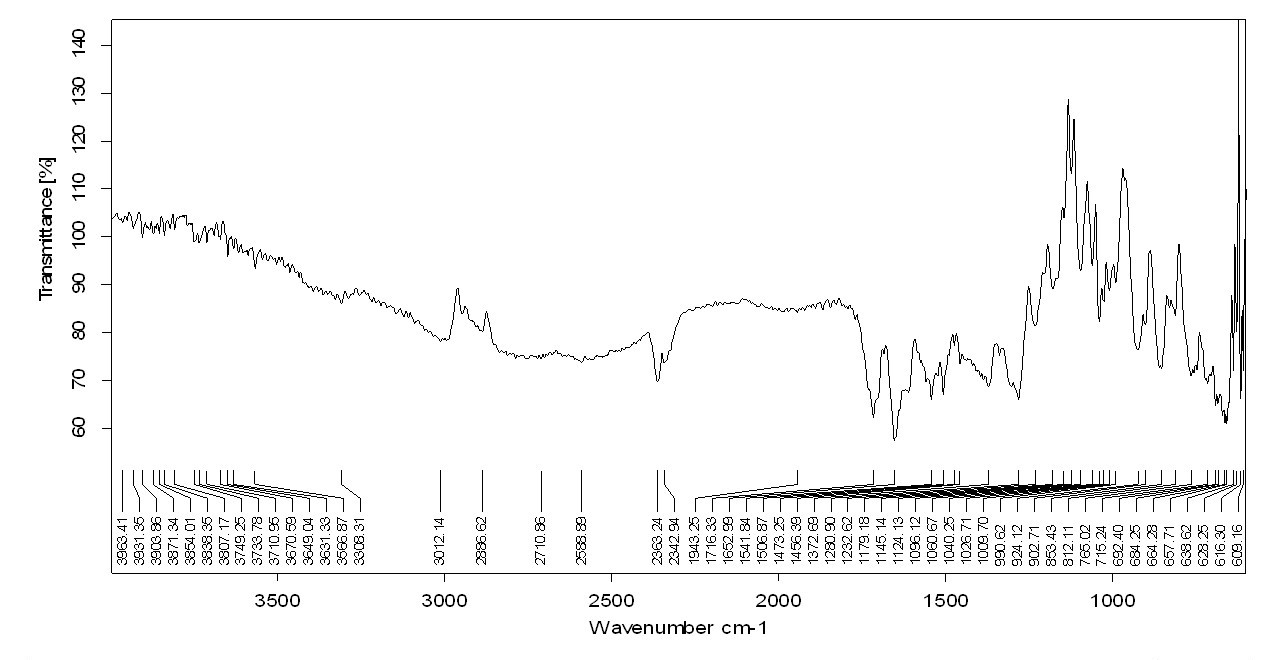


Figure 9. FTIR spectrum of M1


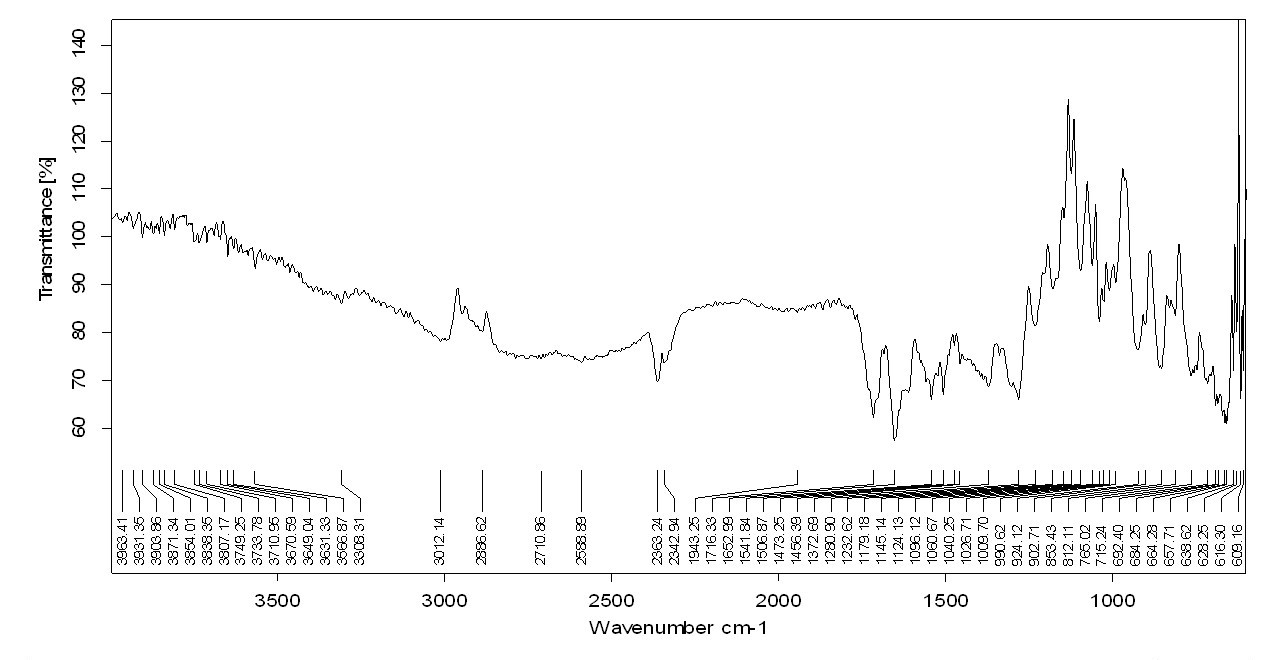


Figure 10. FTIR spectrum of M2


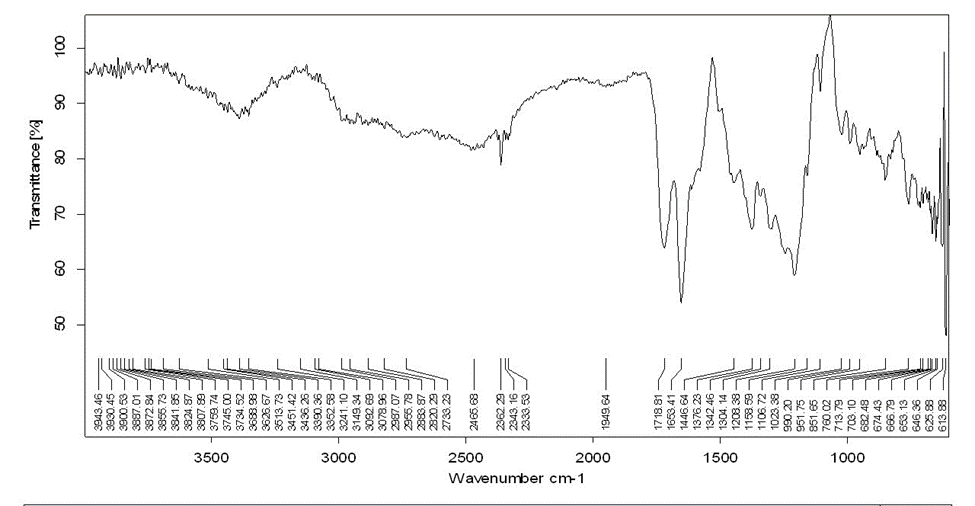


Figure 11. FTIR spectrum of M3


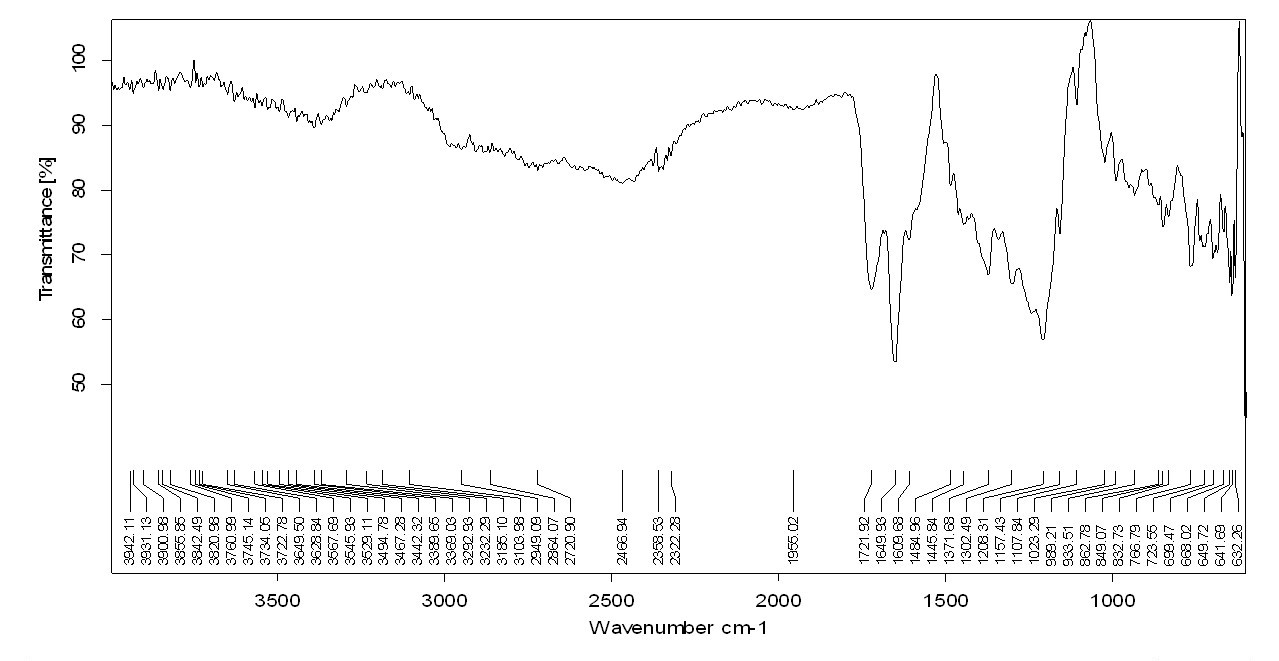


Figure 12. FTIR spectrum of M4


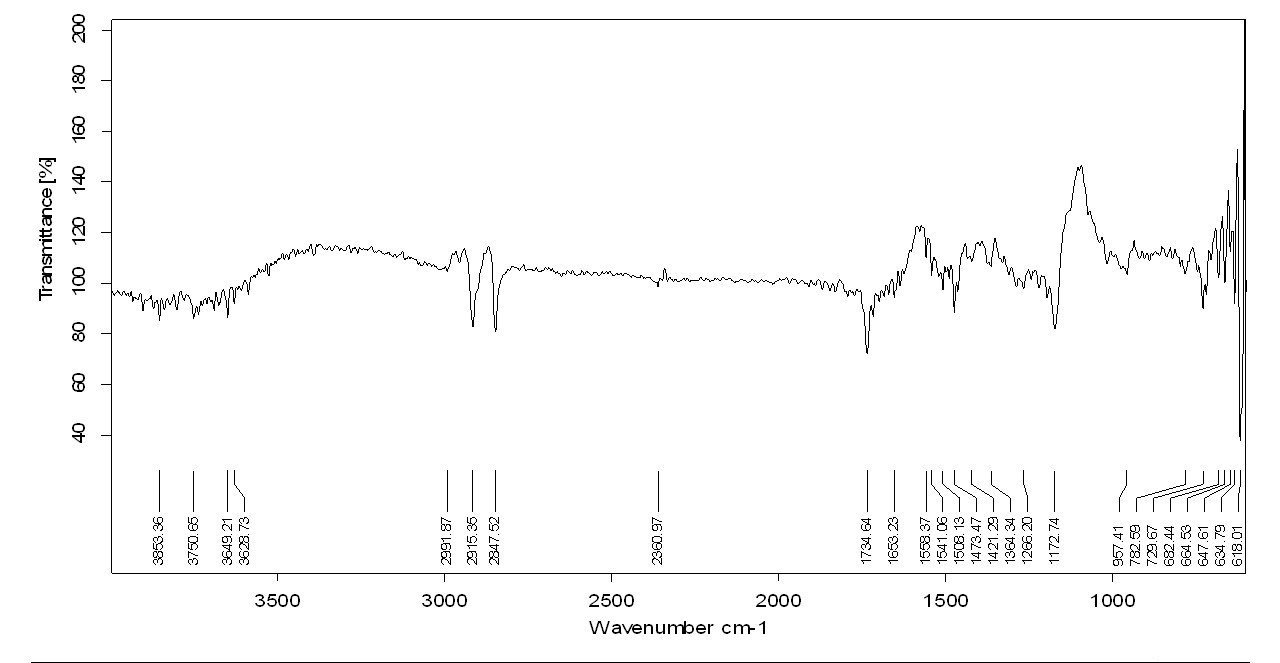


Figure 13. FTIR spectrum of M5


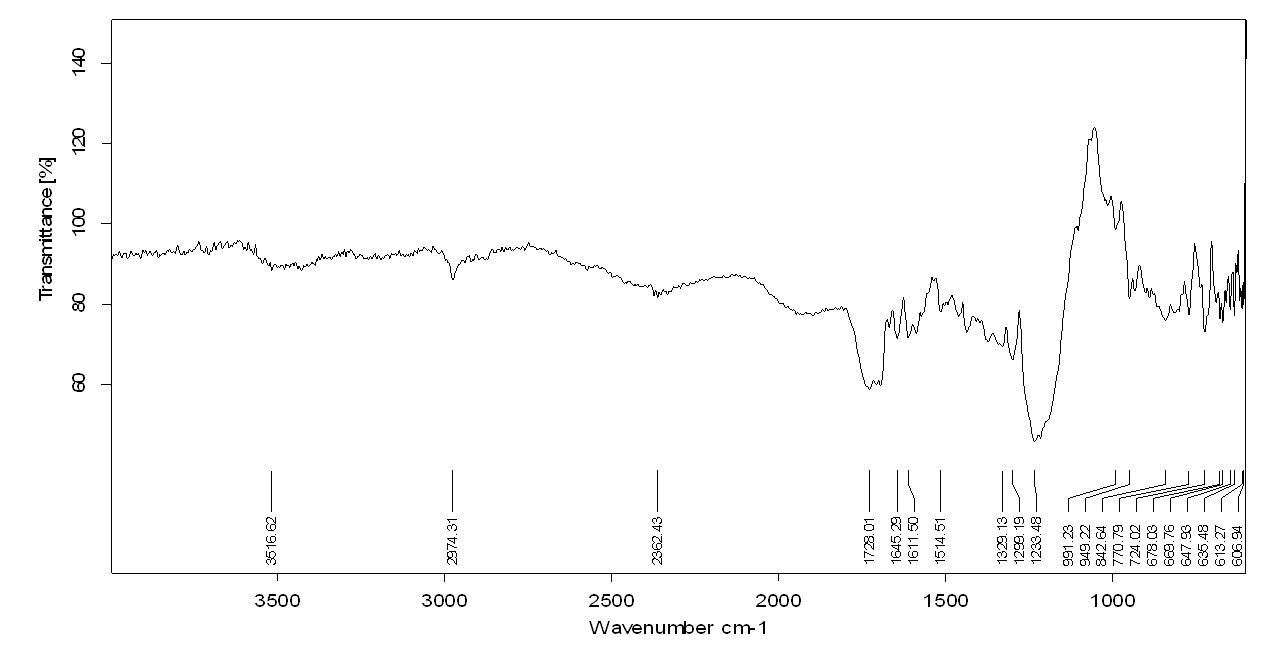


Figure 14. FTIR spectrum of M6


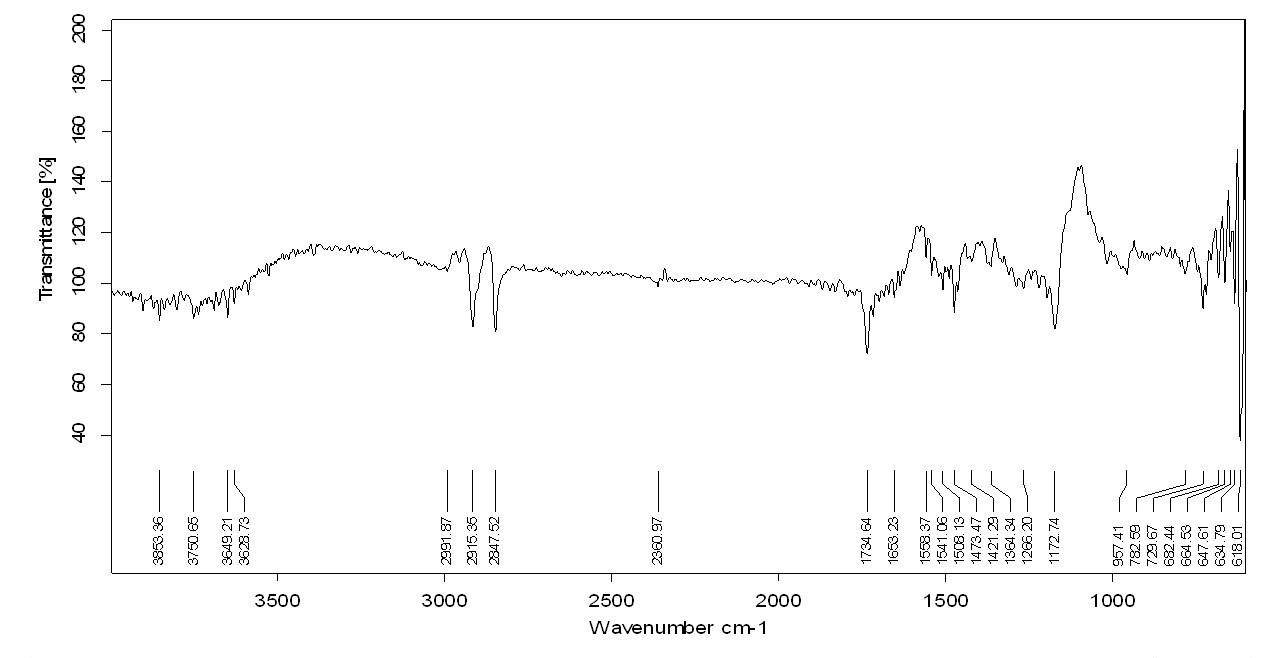


Figure 15. FTIR Spectrum of M7


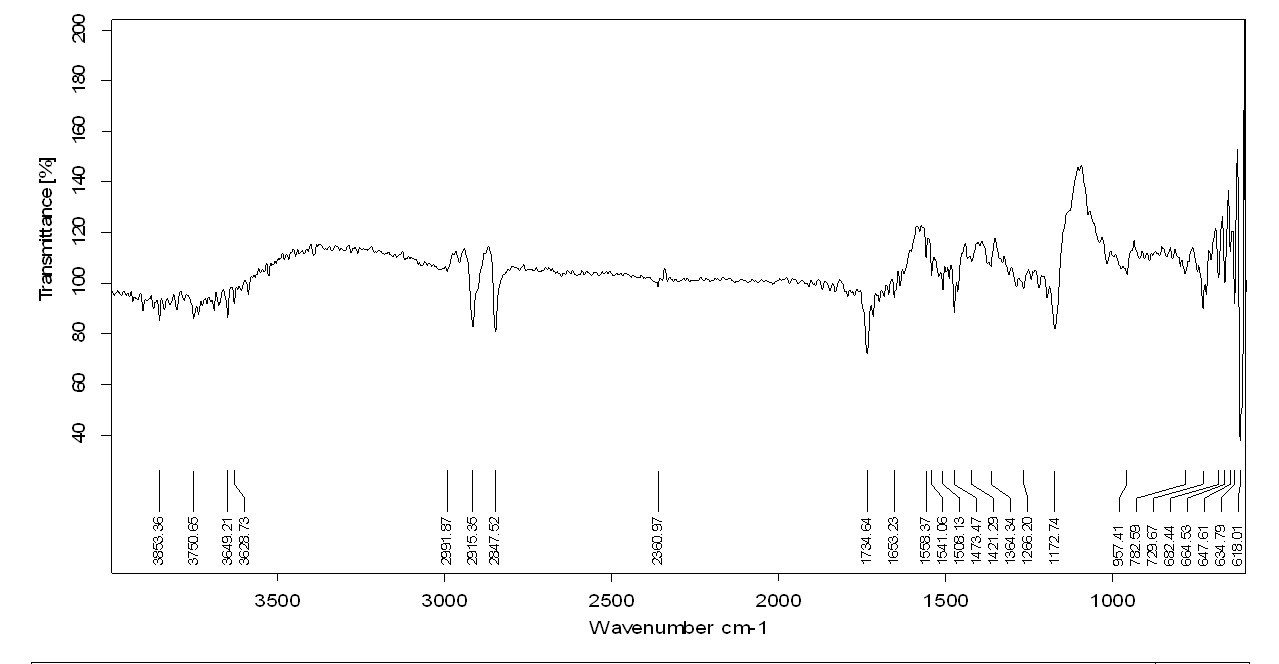


Figure 16. FTIR Spectrum of M8

Figure 17. ^1^HNMR of meropenem

Figure 18. ^13^C NMR of Meropenem

Figure 19. ^1^H NMR of M1

Figure 20. ^13^C NMR of M1

Figure 21. ^1^H NMR of M2

Figure 22. ^13^C NMR of M2

Figure 23. ^1^H NMR of M3

Figure 24. ^13^C NMR M3

Figure 25. 1H NMR of M4

Figure 26. ^13^C NMR of M4


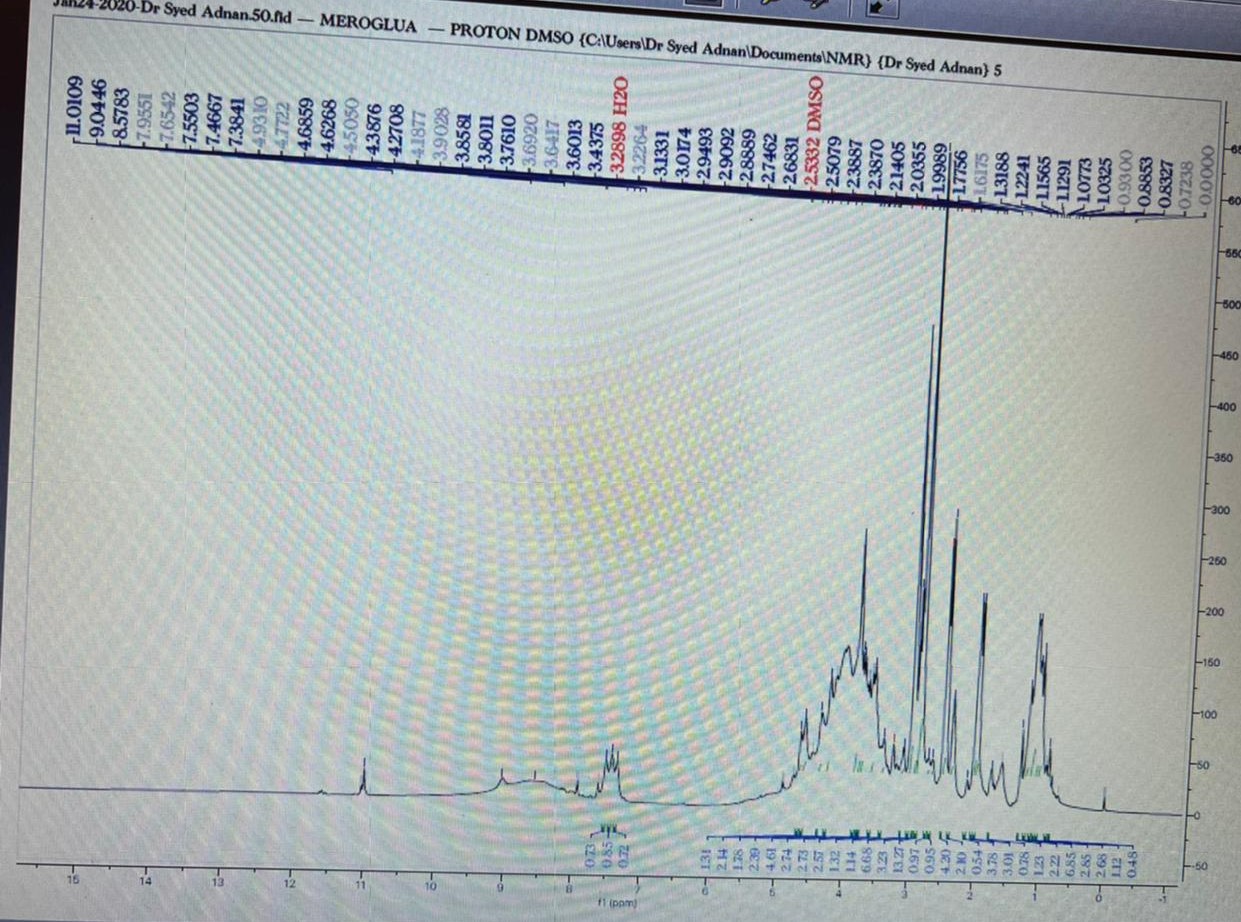


Figure 27. ^1^H NMR of M5

Figure 28. 13C NMR of M5

Figure 29. 1H NMR of M6

Figure 30 13C NMR of M6


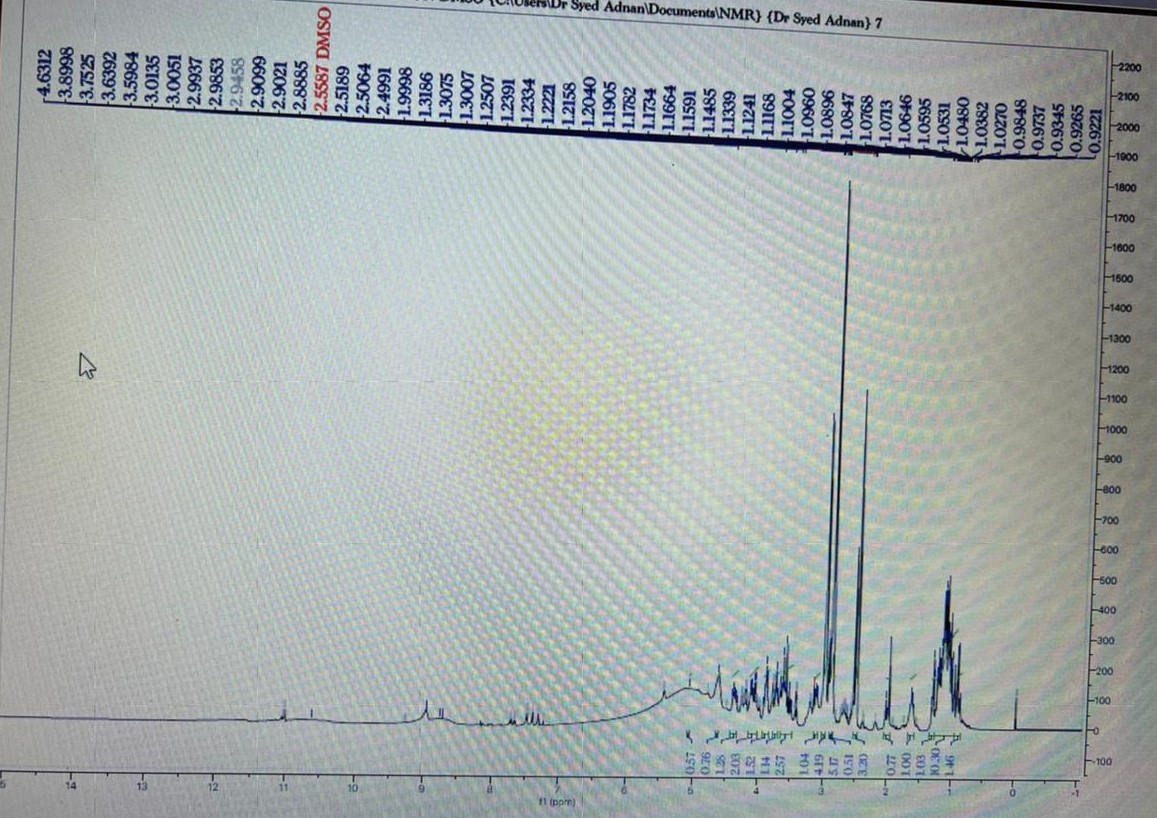


Figure 31. 1H NMR of M7


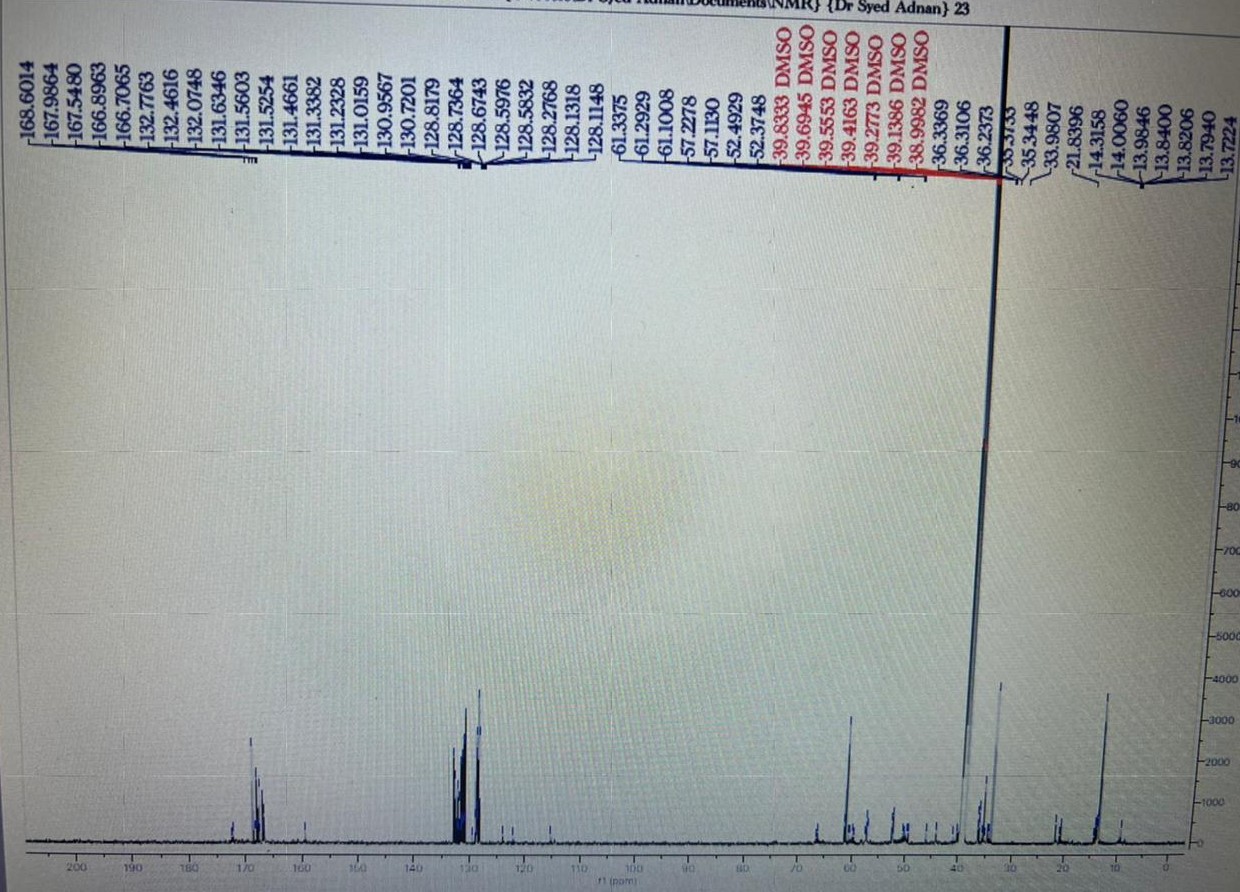


Figure 32. ^13^C NMR of M8


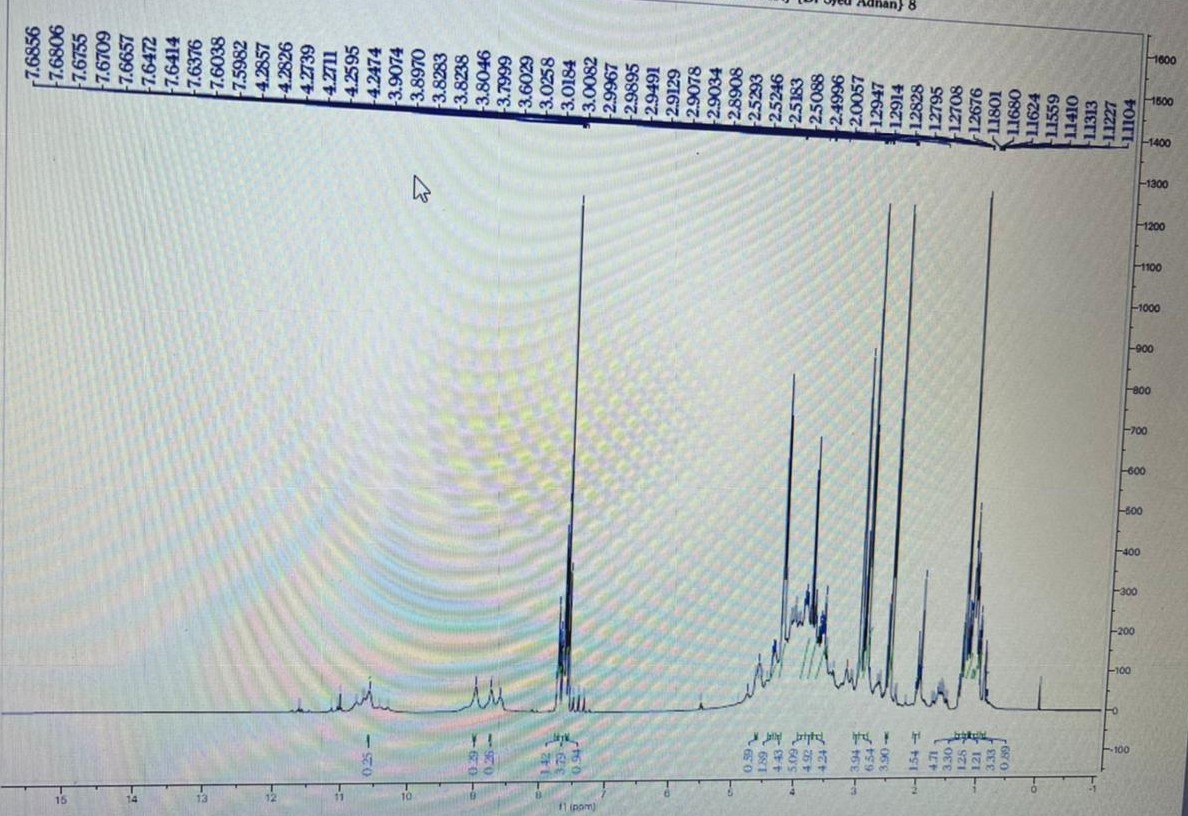


Figure 33. ^1^H NMR of M8
